# Molecular Pathway Reconstruction and Analysis of Disturbed Gene Expression in Depressed Individuals Who Died by Suicide

**DOI:** 10.1371/journal.pone.0047581

**Published:** 2012-10-22

**Authors:** Vladimir Zhurov, John D. H. Stead, Zul Merali, Miklos Palkovits, Gabor Faludi, Caroline Schild-Poulter, Hymie Anisman, Michael O. Poulter

**Affiliations:** 1 Molecular Brain Research Group, Robarts Research Institute, University of Western Ontario, London, Ontario, Canada; 2 Laboratory for Neuromorphology, Hungarian Academy of Sciences and Semmelweis University, Budapest, Hungary; 3 Semmelweis University Hospital, Budapest, Hungary; 4 University of Ottawa Institute of Mental Health Research, Ottawa, Ontario, Canada; 5 Departments of Psychology, Psychiatry and Cellular and Molecular Medicine, University of Ottawa, Ottawa, Ontario, Canada; 6 Department of Neuroscience, Carleton University, Ottawa, Ontario, Canada; Nathan Kline Institute for Psychiatric Research and New York School of Medicine, United States of America

## Abstract

Molecular mechanisms behind the etiology and pathophysiology of major depressive disorder and suicide remain largely unknown. Recent molecular studies of expression of serotonin, GABA and CRH receptors in various brain regions have demonstrated that molecular factors may contribute to the development of depressive disorder and suicide behaviour. Here, we used microarray analysis to examine the expression of genes in brain tissue (frontopolar cortex) of individuals who had been diagnosed with major depressive disorder and died by suicide, and those who had died suddenly without a history of depression. We analyzed the list of differentially expressed genes using pathway analysis, which is an assumption-free approach to analyze microarray data. Our analysis revealed that the differentially expressed genes formed functional networks that were implicated in cell to cell signaling related to synapse maturation, neuronal growth and neuronal complexity. We further validated these data by randomly choosing (100 times) similarly sized gene lists and subjecting these lists to the same analyses. Random gene lists did not provide highly connected gene networks like those generated by the differentially expressed list derived from our samples. We also found through correlational analysis that the gene expression of control participants was more highly coordinated than in the MDD/suicide group. These data suggest that among depressed individuals who died by suicide, wide ranging perturbations of gene expression exist that are critical for normal synaptic connectively, morphology and cell to cell communication.

## Introduction

In an effort to understand the biological processes associated with depression and suicide, one viable approach has been the molecular analysis of brain tissue obtained from depressed individuals who died by suicide relative to non-depressed controls who died from causes other than suicide. In this regard, marked differences have been shown with respect to the expression of CRH, 5-HT and GABA_A_ receptor subunits mRNAs and protein between depressed and non-depressed individuals [1]–[3].

The use of microarrays as a means of “gene discovery” has provided novel insights into various groups or subgroups of genes that may be associated with depression/suicide [4]–[6]. The significance or meaningfulness of the altered expression of a gene has relied upon the researcher understanding the functional implications of these genes. At another level, ontology lists can be created that might suggest how a set of genes might operate together to determine more complex phenotypes. For example, a gene list that included down-regulated genes that control cell differentiation might implicate impaired development of a normal phenotype. Beyond this level of analysis, considerable difficulty can be encountered in the interpretation of microarray data as the functional implications of hundreds of gene changes is reliant on the end user having broad knowledge of all potential protein/protein interactions that could be altered.

One (potential) solution to this inability to analyze gene sets rationally has come from the use of software that “reads” vast amounts of information (e.g., from PubMed) and then constructs relationship maps that permit the user to identify known or potential novel processes that may be altered. Following from this method, in the present study we used microarrays to compare the mRNA expression of frontopolar cortex, a region implicated in depression and suicide [7], [8], obtained from control and depressed/suicide subjects. We then implemented a method of analysis that “reads” the current medical literature, thus permitting the construction and display of relationships between various biological molecules and processes. This analysis implicated a number of processes involved in cell to cell adhesion and brain structural processes that appear to be perturbed in the depressed/suicide brain. Since this analysis provides evidence for the functional interactions between all gene products, it is also able to point out potential functional “hubs” where one protein may be central in the functioning of many others. This approach to understanding the involvement of gene sets or hubs in relation to pathology has been used in the analyses of cancer [9]–[11] and to our knowledge it has not previously been adopted for brain related disturbances. To be sure, when multiple relations are conducted, even when premised on the scientific literature, the risk of alpha error is exceedingly high. However, it should be no greater in control than in brain tissue obtained from depressed individuals that died by suicide. Thus, this approach, despite its inherent limitations with respect to any one gene, provides important clues regarding network differences that might exist between these groups.

## Materials and Methods

### Description of Subjects Analysed

Gene expression was analyzed from non-psychiatric control subjects (N = 9) and depressed individuals who died by suicide (N = 10). All subjects were Caucasian Hungarian males. Tissue samples were obtained at autopsy at the Department of Forensic Medicine of the Semmelweis University Medical School in Budapest (as described in [8]). The suicide and control groups were of approximately equal age, had similar brain pH, post mortem interval (PMI) and RNA quality (as measured by RNA integrity number; RIN) (see Table 1 for summary). Cause of death is also listed in Table 1.

**Table 1:** Summary of the attributes of the cohort of subject used for analysis.

Control	Cause of Death	Age	RIN	PMI	Brain pH
1	AMI	56	7.2	2	6.62
2	AMI	46	5.5	4	6.36
3	ACF	67	5.2	1	6.35
4	ACF	45	6.6	5	6.43
5	ACF	49	6.9	6	6.15
6	ACF	41	5.2	2	6.22
7	ACF	75	6.9	1	6.79
8	ACF	73	8.5	6	6.96
9	ACF	83	6.9	6	6.74
	**Average**	**59.4**	**6.5**	**3.7**	**6.51**
	**SEM**	**4.7**	**0.4**	**0.8**	**0.10**
**Depressed Suicide**					
1	Hang	62	6.3	2.5	6.63
2	Hang	42	5.7	3	6.51
3	Hang	45	5.7	4	6.77
4	Hang	47	6	6	6.92
5	Hang	55	8.5	4	6.97
6	OD	49	8.3	6	6.59
7	Hang	49	8.6	4.5	6.45
8	Jump	71	8.3	1	6.64
9	Hang	48	8.6	6	6.28
10	Hang	57	8.4	16	6.58
	**Average**	**52.5**	**7.4**	**5.3**	**6.63**
	**SEM**	**2.8**	**0.4**	**1.3**	**0.07**
	**p value**	**0.46**	**0.13**	**0.25**	**0.22**

Abbreviations used myocardial infarction; MCI; Acute cardiovascular failure: ACF Hang; death by hanging; jump death by jump form a height; over dose; OD.

Tissue harvesting occurred after written informed consent was obtained from next of kin, which included the request to consult the medical chart and to conduct neurochemical and/or biochemical analyses. The ethics committee at Semmelweis and the Ethics Committees of Carleton University and the University of Western Ontario approved harvesting and analyses of the tissue samples. The ethical rules for dissecting human brains vary across countries. In some of the European countries, as in Hungary, once death is confirmed by 3 physicians/pathologist, the removal of the brain may proceed. In the cases of persons who died by suicide or in traffic accident, pathological sectioning, as “medicolegal cases”, is ordinarily obligatory. These brains may be removed from the skull as soon as 1–2 hours post mortem, frozen and stored until the pathological sectioning. The dissection (microdissection) of the brain can be performed after pathological diagnosis has been obtained, including tests for HIV, tuberculosis, syphilis, hepatitis, presence of alcohol and other drugs.

The suicide condition comprised individuals that died by hanging, drug overdose or jump from height. Medical, psychiatric and drug history of suicides were obtained through chart review coupled with interviews with the attending physician/psychiatrist and family members, as previously described [8]. These interviews were semi-structured and focused on issues such as previous psychiatric history, family history of mental illness, and recent stressful experiences. In each instance a psychiatric diagnosis of depressive disorder was previously on record. The diagnoses were conducted and/or confirmed by experienced psychiatrists on the basis of DSM-IV criteria. Insofar as could be determined, the participants had not used antidepressant medication for at least two months prior to death and did not have a history of either drug or alcohol abuse. Toxicological tests of blood samples confirmed that drugs or alcohol were not present in cases of death by hanging or jump from height.

With respect to the control participants, examination of medical records confirmed the absence of a history of psychiatric illness, alcohol or drug abuse during the last ten years. Moreover, interviews with family members indicated that control participants had never been treated for depression, and did not have a history of alcohol abuse. Causes of death in control subjects were acute cardiac failure, myocardial infarction or traffic accident. In all instances death was sudden and did not involve a prolonged agonal state.

### Tissue Collection, Dissection and Storage

Brains were obtained 1–6 hours after death in Budapest, Hungary. After removal from the skull, the brains were cut in six major pieces (four cortical lobes, basal ganglia-diencephalon, and lower brainstem-cerebellum), rapidly frozen on dry ice, and stored at −80°C until dissection (which occurred 2 days to 2 months later). At the time of the dissection, the brain samples were sliced into 1 to 1.5-mm-thick coronal sections at a temperature of 0–10°C. Cortical samples were always taken from the right hemisphere. The frontopolar (FPC) region was cut out of the sections by a fine microdissecting (Graefe’s) knife. This comprised Brodmann area 10, dissected at the most polar portion of the frontal lobe below the intermediate frontal sulcus. The samples were stored in airtight containers or plastic tubes at −80°C until use. RNA was extracted using Trizol reagent (Invitrogen, Carlsbad, California). RNA quality assessment was performed using Agilent 2100 Bioanalyzer (Agilent Technologies, Santa Clara, California). Table 1 provides a description of the brain pH, post mortem interval and RNA integrity number (RIN) for each sample and cause of death. None of these variables were found to differ significantly between the controls and depressed individuals that died by suicide (p>0.05).

### Microarray Experiment

We utilized GeneChip Human Genome U133 Plus 2.0 Array (Affymetrix, Santa Clara, California), which analyzes expression level of over 47,000 transcripts, including 38,500 well-characterized human genes.

### Data Analysis

MAS5 probe level expression data generation algorithms were used as implemented in Affymetrix Expression Console software version 1.1. Expression data were filtered using MAS5 detection call with threshold of ≥50% present in both classes [12]. If a gene was considered to be present it was assigned the value of 1, a marginal presence was given a value of 0.51 and an absent call was assigned a value of 0. For a probe set to be considered for subsequent analysis the sum of call values from each subject had to exceed 4.59 for the control group (n = 9) and 5.10 (n = 10) for the suicide group.

#### Power analysis and FDR assessment

Partek Genomics Suite (GS) (Partek, St. Louis, Missouri) was used to determine differentially expressed genes between depressed suicide patients and non-psychiatric controls using Principal Component Analysis (PCA) and Analysis of Covariance (ANCOVA). We have used subject’s age, brain pH, PMI and RIN as covariate factors in ANCOVA. Effect of these covariate factors was removed from the data set using batch remove tool of Partek GS. Probe sets which demonstrated significantly different expression levels between classes at *p*<0.01 with Fold Change (FC) >1.3 in either direction were considered for subsequent analysis.

We performed *post-hoc* power analyses of the mRNA expression data at α = 0.01, β = 0.2 and a Fold Change |FC| = 1.3 cut-off using the interactive power analysis tool for microarrays HCE 3.5 [13]. The analyses showed that sufficient power existed to detect differentially expressed genes at these cutoffs.

We found traditional FDR control methodologies such as BH [14] to be too conservative for our data set after removal of covariates’ effect. However, we performed an assessment of FDR using Significance Analysis of Microarrays (SAM) [15] workflow which demonstrated that with our *p* and FC cut-offs FDR is controlled at approximately 0.01.

The probe sets representing 238 known genes, obtained by filtering expression data according to criteria described earlier, were loaded into Pathway Studio software version 6.2 (Ariadne Genomics, Rockville, Maryland) pathway analysis. Pathway Studio software builds and displays molecular pathways and connections of biomedical interest. It allows for interpretation of experimental results in the context of pathways, gene regulation networks, and protein interaction maps. When performing pathway reconstruction in Pathway Studio software it is important to note that reported relationships are not necessary direct in the biochemical or protein interaction sense. What is reported is an implied causal relationship extracted from existing scientific literature. Gene interaction networks were generated to show known direct relationships involving differentially expressed genes in the data set. Small networks of less than four proteins were manually discarded.

To determine whether generated network represent a true biological difference between two classes, we generated 100 random probe sets chosen from all genes that were considered to be present on the arrays. The lists of these sets were used to reconstruct relationship networks and then were assessed for complexity (the number of proteins and relationships within the generated networks). A bootstrapping analysis was then performed to determine whether the networks generated from the differentially expressed gene sets could be considered to be part of the randomly generated networks. In this regard, a Z score of >3.0 was considered to be statistically significant.

To further analyze differentially expressed genes with known relationships, we generated gene networks based on Pearson correlations for both classes using Pathway Studio software. We used a *p*-value cut-off of 0.01, Pearson’s correlation (r) cut-off of 0.8, removal of 5% of genes with the most stable expression and only the largest gene networks were considered for subsequent analysis.

To identify the possible function of the gene lists generated by the Pathway studio analysis, we also performed functional annotation and clustering GO analysis using DAVID (ver 6.7). Gene lists that were analyzed were the following: differentially expressed genes (238 genes); differentially expressed genes involved in direct relationships (46 genes); differentially expressed genes with correlated expression in the control group (45 genes); differentially expressed genes with correlated expression in the suicide group (21 genes).

### QPCR Validation

Samples for Quantitative PCR (QPCR) analyses were prepared by reverse transcribing 3 µg of total RNA using Superscript II reverse transcriptase (Invitrogen Canada, Burlington, ON). Aliquots of this reaction were then used in simultaneous QPCR reactions. RNA extraction and QC was performed in the same way as described for microarray experiment.

For QPCR, SYBR Green detection was used according to the manufacturer’s protocol (iQ SYBR Green Supermix; Bio-Rad, Hercules, CA). A Bio-Rad MyiQ real-time thermocycler was used to collect the data. All of the PCR primer pairs used generated amplicons between 90 and 120 bp. Primer efficiency was measured from the serial dilutions of cDNA over the range that incorporated experimental cDNA amounts using iQ software. All of the primer pairs had a minimum of 90% efficiency. We choose 10 genes at random over the range of expression of FC 0.5 to 2.5.

These genes were as follows: β2 microglobulin, B2M, calreticulin, CALR, caveolin 1, CAV1, caveolin 2, CAV2, coronin 2A, CORO2A, glutamine synthetase, GLUL, lumican, LUM, neuronal cell adhesion molecule, NRCAM, prion protein, PRNP, sorting nexin 2, SNX2, RNA polymerase II polypeptide A, POLR2A. Primers that amplify RNA polymerase II mRNA were used as a reference gene to normalize the data. (PCR primer sequences can be found in Supplemental data).

## Results

**Figure 1:**
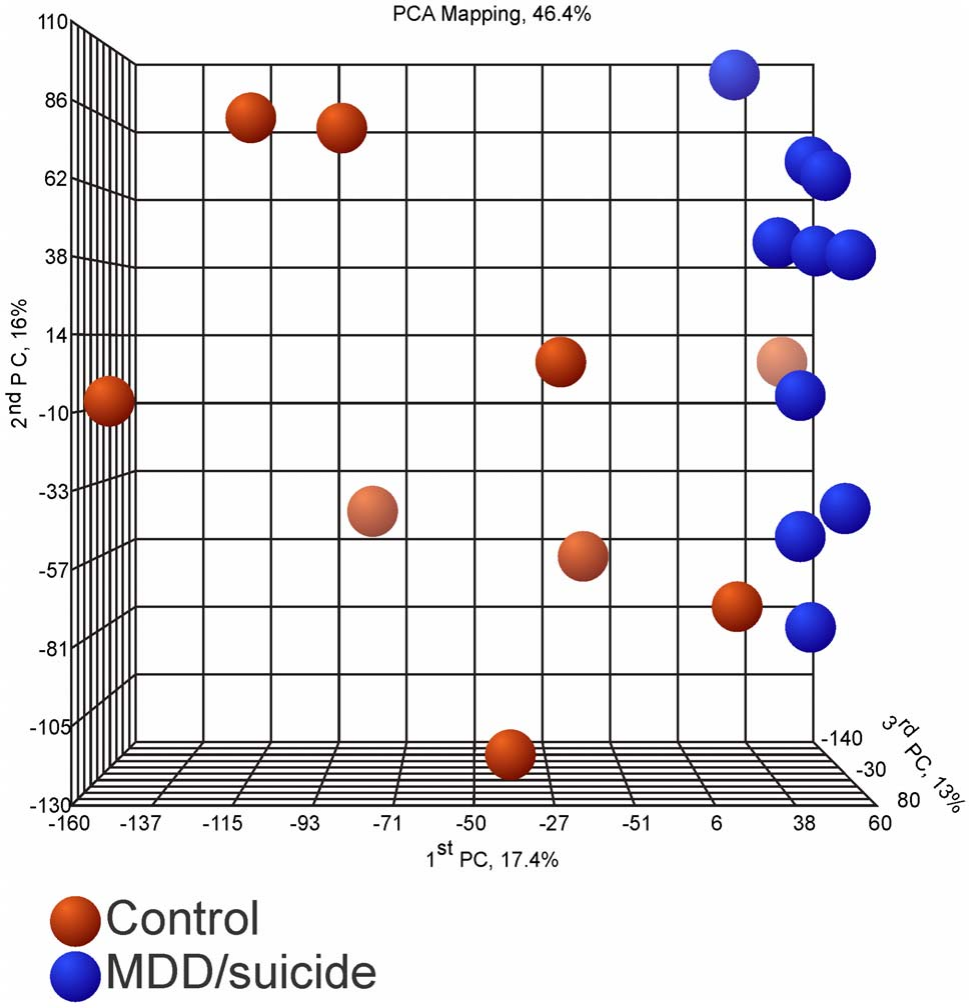
PCA demonstrates separation of control and depressed suicide subject groups. PCA of microarray expression data based on complete data set. Red nodes represent control subjects, blue nodes - depressed suicide victims. Variable shading indicates distance from a viewer in 3D space.

**Figure 2:**
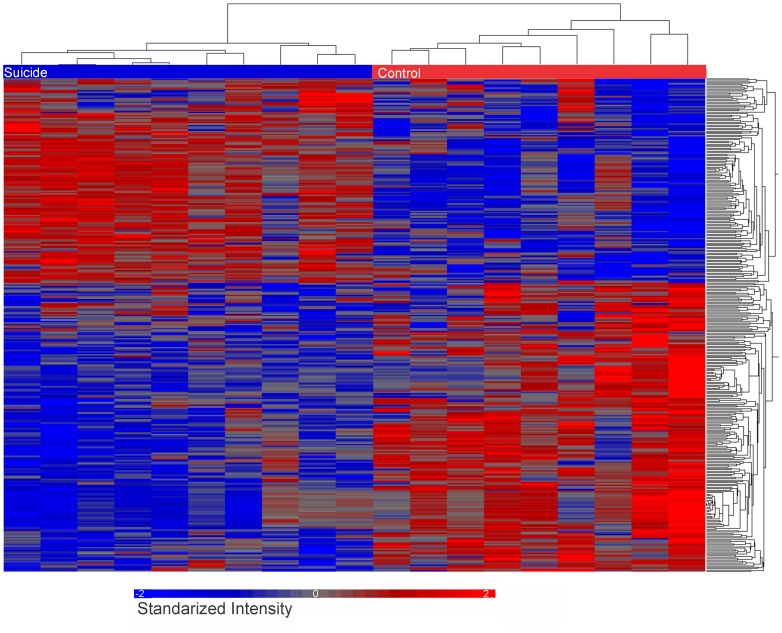
Heat Map and dendrogram of all Control and depressed/suicide samples show both up and down-regulation and clustering of 238 differentially expressed genes. Relative Expression values were normalized across all samples within each data set. Rows represent probes while columns represent individual samples. Grey bars indicate no difference in expression, whereas blue and red indicate more and less expression, respectively.

In order to determine whether the gene expression of two groups was different, principal component analysis (PCA) was performed on the complete P/A call-filtered MAS5 data set (as implemented in the Partek Genomics Suite). The first three principal components of this data set explained 46.4% of the variability between control and depressed/suicide classes and demonstrated a separation of classes, albeit with some overlap (Figure 1). This analysis also showed higher variability of gene expression in the control than in the depressed suicide group. At fold change (FC) of 1.3, we found 340 differentially expressed probe sets, *p*<0.01. Figure 2 shows a ‘heat map” representation of the differentially expressed genes (DEGs) found in this analysis. These 340 probe sets corresponded to 238 annotated genes. Gene ontology analysis of the 238 DEG showed that it was enriched with genes involved in intracellular protein transport, synaptic transmission and cell-cell signaling, *p’s* <0.01 (Table 2, Table S1).

**Table 2:** Summary of GO cluster analysis results for the lists of all differentially expressed genes.

Differentially Expressed Genes		
GO Term	Gene count	*p*
cellular homeostasis and signaling	158	4.8E−05
synaptic transmission	14	1.2E−04
cellular localization	27	1.3E−04
transmission of nerve impulse	15	1.7E−04
localization of protein	60	2.2E−04
homeostatic process	22	6.4E−04
establishment of cellular localization	53	7.3E−04
transport	52	1.0E−03
establishment of localization in cell	23	1.3E−03
cell-cell signaling	18	1.8E−03

Enrichment score is a –Log10 of a geometric mean of individual *p* reported for individual GO terms within cluster. Only the top two clusters that exceed enrichment score cut-off of 2 (corresponds to geometric mean of *p* = 0.01) are listed. The listed terms that are present in cluster are the most representative of the main themes of all GO terms included in cluster.

**Figure 3:**
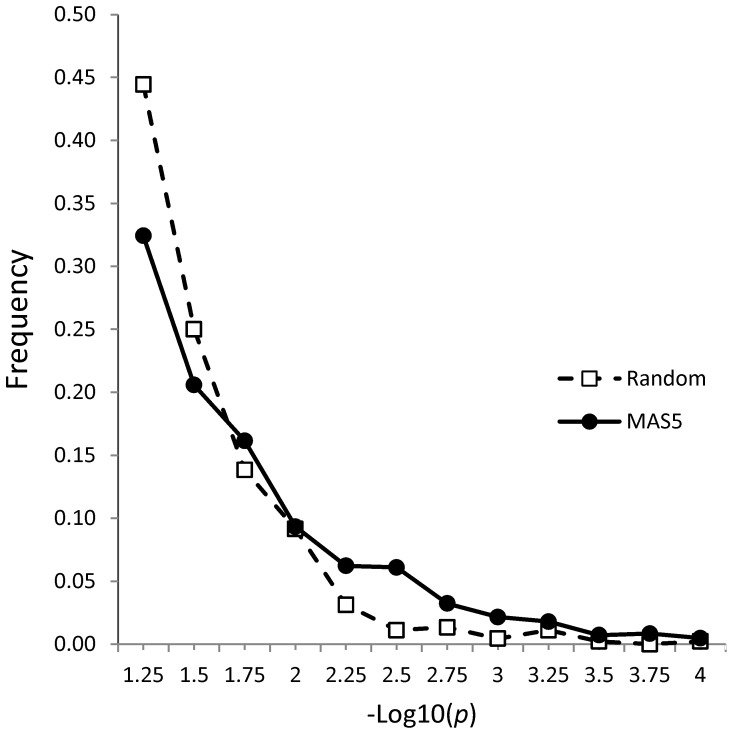
Functional annotation analysis demonstrates that differentially expressed probe sets represent true biological difference between control and depressed suicide subject groups. Comparison of functional annotation analysis of differentially expressed probe sets generated at different *p* and FC cut-offs and randomly generated probe sets lists of the same size. Note the shift of the experimental curve to the right.

To assay whether differentially expressed probe sets represented a true biological difference between two groups, we generated a number of probe set lists at different *p* and FC cut-offs and compared them to randomly generated probe set lists of the same size using DAVID (Database for Annotation, Visualization and Integrated Discovery) functional annotation analysis [16]. We generated nine differentially expressed probe set lists at combinations of *p* equal 0.001, 0.01 and 0.05 and FC equal 1.3, 1.4 and 1.5 cut-offs. A number of random probe set lists of the same size were then generated from the complete probe set list. Functional annotation analysis was conducted using DAVID and frequency distributions of GO enrichment scores were compared. The frequency plot of the MAS5 generated gene set was not the same as a similar plot where a random gene list was generated (Figure 3). Importantly, the random list had maximum -log *p* values of 3.8, whereas, the maximum -log *p* value obtained from our dataset was 5.8. Thus our differentially expressed gene list cannot be considered to be a random outcome.

**Figure 4:**
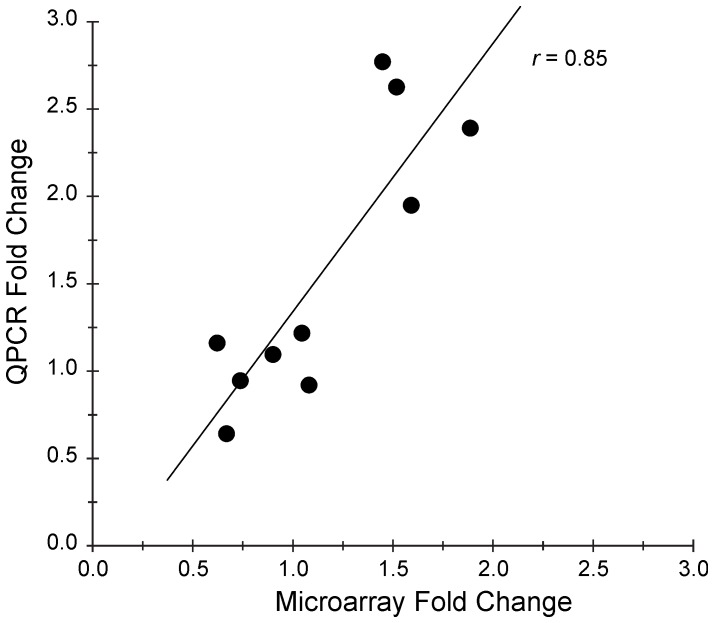
Relationship between Fold Change (FC) reported in microarray and QPCR experiments. Pearson’s correlation r = 0.86 and *p*<0.01. Circles represent individual genes. If more than one probe set was present in microarray dataset for a particular gene, an average FC was used for this gene as a “MAS5 FC”.

This list was further validated by performing QPCR analysis on a subset of genes. This analysis demonstrated high agreement between microarray and QPCR results (Figure 4). The fold change found in microarray experiment was positively correlated with the fold change found in the QPCR experiment (*r* = 0.85, *p*<0.01).

### Pathway and GO Analysis

**Figure 5:**
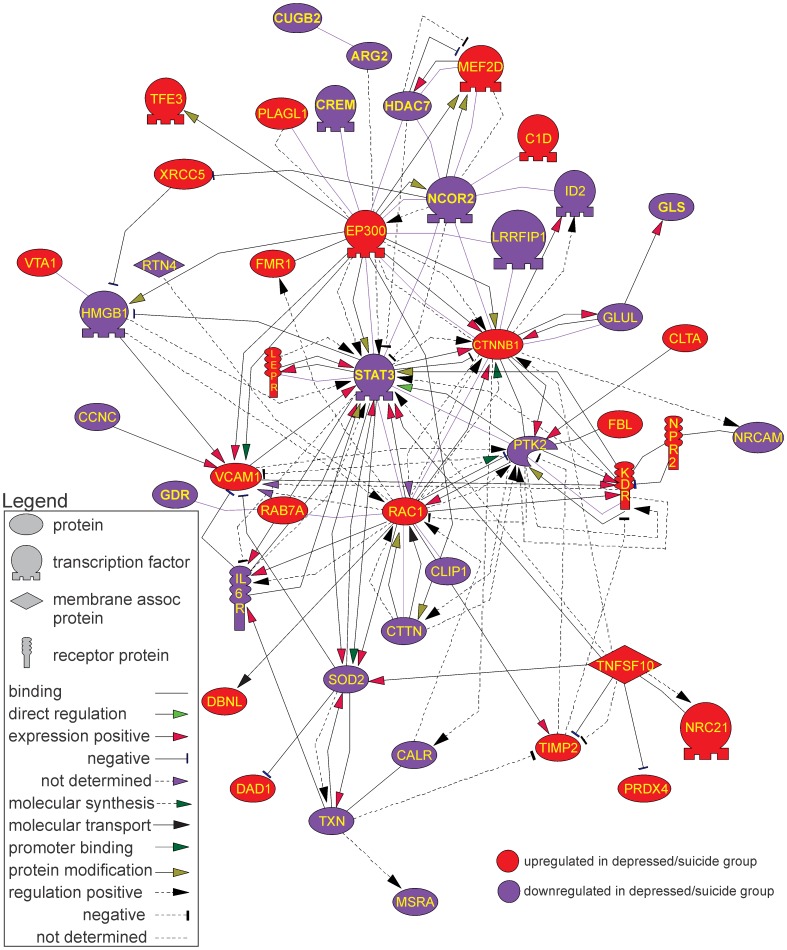
Gene relationship network generated for differentially expressed genes based on known direct relationships. For detailed description of various types of relationships see Table S1.

The potential biological differences that may occur between differentially expressed genes (DEGs) was determined by performing an analysis that interprets how the DEGs are known to interact in biological pathways, gene regulation networks, and protein interaction maps. This analysis, termed pathway reconstruction, computationally finds how/if gene products have previously reported functional relationships based on what is known from existing scientific literature (see Methods for complete description). This analysis generated 7 pathways utilizing 59 proteins that had 165 functional relationships. However 6 of the 7 pathways involved 4 or fewer proteins, and were thus not considered further. The seventh network comprised 46 proteins with 157 known direct relationships (Figure 5).

The shape of each symbol representing a gene indicates the putative function of the gene product. In addition the figure indicates, by colour, whether the gene was up-regulated (red) or down-regulated (blue). Functional annotation and enrichment analysis of these 46 proteins demonstrated that this pathway was enriched (p<0.01) with gene products involved in cell differentiation, neurogenesis and axon growth (Table 3). Interestingly, five gene products appeared as network “hubs” as they were functionally implicated with at least 20 other proteins (see Figure 5 and Table 4). Each of these hubs is a gene that has an established functionality in the central nervous system.

**Table 3:** Summary of GO cluster analysis results for the lists of differentially expressed genes with known direct relationships.

Genes with direct relationships			
GO terms present in cluster	Enrichment score p value range	Genes present in cluster	Gene Count
positive regulation of cellular andbiological process, systemdevelopment	4.0 1E−04<p<0.01	CALR, CTNNB1, EP300, FMR1, HDAC7, ID2, IL6R, KDR, LEPR, MEF2D, NRCAM, NRP2, PLAGL1, PTK2, RAC1, RTN4, SOD2, STAT3, TFE3, TIMP2, TNFSF10, VCAM1, XRCC5	23
regulation of cellular and biologicalprocess	3.9 1E−04<p<0.001	ARG2, C1D, CALR, CCNC, CREM, CTNNB1, DAD1, DBNL, EP300, GDI1, HDAC7, HMGB1, ID2, IL6R, KDR, LEPR, LRRFIP1, MEF2D, NCOR2, NR2C1, NRCAM, PLAGL1, PRDX4, PTK2, RAB7A, RAC1, RTN4, SOD2, STAT3,TFE3, TIMP2, TNFSF10, TXN, VCAM1, XRCC5	35
regulation of cell differentiation and developmental process	3.9 1E−06.<p<0.01	CALR, CTNNB1, HDAC7, ID2, IL6R, NRCAM, PTK2, RTN4, SOD2, TFE3, TIMP2, XRCC5	12
neurogenesis, cell differentiation, developmental process, cell development	3.7 1E−04<p<0.01	ARG2, CALR, CREM, CTNNB1, DAD1, EP300, FMR1, ID2, IL6R, KDR,LEPR, MEF2D, NRCAM, NRP2, PTK2, RAC1, RTN4, SOD2, STAT3,TIMP2, VCAM1, XRCC5	22
regulation of cell differentiation, neurogenesis, regulation of axiogenesis	3.3 1E−04<p<0.06	CALR, CTNNB1, EP300, HDAC7, HMGB1, ID2, IL6R, KDR, NRCAM, NRP2, PTK2, RAC1, RTN4, SOD2, STAT3, TFE3, TIMP2, XRCC5	18

Enrichment score is a –Log10 of a geometric mean of individual *p* reported for individual GO terms within cluster. Only top 5 clusters that exceed enrichment score cut-off of 2 (corresponds to geometric mean of *p* = 0.01) are listed. The listed terms present in cluster are the most representative of the main themes of all GO terms included in cluster.

**Table 4:** Summary of the known functions of genes that were found to be “hubs” in the pathway analysis network of DEGs’.

Gene symbol	Gene name	Role
CTNNB1	catenin, beta 1	nervous system development, neuroprotection
EP300	E1A binding protein p300	neuronal differentiation
PTK2	protein tyrosine kinase 2	neuronal migration, neuronal plasticity
RAC1	rho family, small GTP binding protein	Neuronal development,myelination
STAT3	signal transducer and activator of transcription 3	neuronal survival and regeneration, leptin signalling

In order to further validate this assessment a pathway analysis was performed where random 238 probes sets were selected from all genes shown to be present. This was done 100 times. For each of these randomly chosen gene lists the same analysis was performed as that used for the gene list found to be differentially expressed. As in the MAS5 generated data set, determinations were made of 1) the total number of networks and the number of proteins and relationships in all pathways; 2) the largest network generated, and 3) proteins and relationships per network. These simulations did not produce networks of similar size nor complexity. This is summarized in Table 5 where we compare the average complexity of these simulations in comparison to the values we found in the MAS 5 generated data set. For each parameter the data set was significantly different from the simulations (*p* values for each <0.01).

**Table 5:** Summary of the attributes of the randomly generated gene networks and the networks that were generated from the MAS5 DEG list.

Parameter	Average for 100 generated lists ± SEM	MAS 5 generated list	Z	p
Proteins	45.6±1.0	59	13.4	<0.01
Relationships	66.3±2.7	165	36.0	<0.01
Proteins in the largest network	29.1±1.4	46	12.5	<0.01
Relationships in the largest network	53.7±3.2	157	32.6	<0.01
Proteins per network	7.0±0.4	8.4	3.6	<0.01
Relationships per network	11.0±0.9	23.6	14.0	<0.01

In comparison to parameters from the randomly generated networks, each parameter from the Mas 5 DEG generated Z-scores that were significantly different from the average values that were generated by 100 simulations.

**Figure 6:**
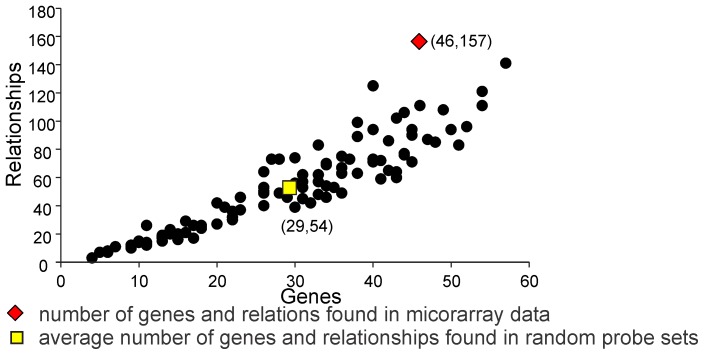
Comparison of complexity of relationship networks generated for the DEG’s list and 100 random probe set lists of the same size. Total number of entities and relationships present in the largest network generated for a list were used to construct the graph. Circles represent random probe set lists, yellow square represents the average for 100 random list networks and the red diamond represents the DEG list. The red triangle shows the value of entities versus relationships found from our gene list. The Z score for this value in relation to average (yellow square) was 12.5 (p<0.01).

The experimental DEGs list generated values that were up to 36 Z-scores from the means generated from the random lists (p<0.01). Specifically, we found that the randomly generated probe lists produced a range of 2–15 network sets (median = 8). The total number of proteins and relationships in these networks ranged from 25–73 and 20–144, respectively, with approximately 46 proteins and 66 relationships on average. Mean connectivity of the largest networks from 100 random probe set lists was 1.7 relationships per entity, whereas the connectivity within the network generated from our experimental data was twice as high (3.4 relationships per entity). The biggest individual network generated from the random gene lists had only 73 proteins with only 95 relationships. In all cases, the number of relationships in the biggest networks generated for each simulation was considerably less than those generated from the experimentally derived DEG list (shown in Figure 5). To further illustrate this difference, Figure 6 shows a plot of the number of proteins in the largest network for each simulation versus the number of relations within each of these networks and the corresponding value from the network. The number of proteins and relationships from the experimentally derived data is graphed as well. As can be seen, these values do not lie within the distribution of the random gene list values. Thus, it is highly unlikely that the network generated from the biological data occurred by chance.

### Correlative Gene Expression Analysis

**Figure 7:**
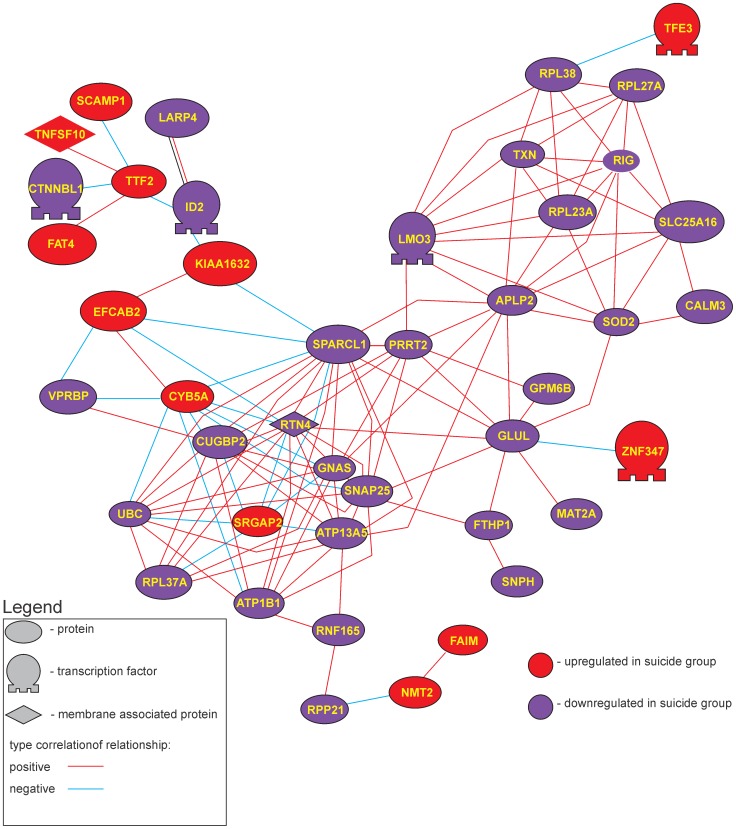
Pearson’s correlation based gene network for differentially expressed genes for the control cohort. Red nodes represent genes up-regulated in the depressed suicide group, blue nodes represent genes down-regulated in the depressed suicide. Network contains 45 genes connected by 134 correlation relationships higher than 0.8.

**Figure 8:**
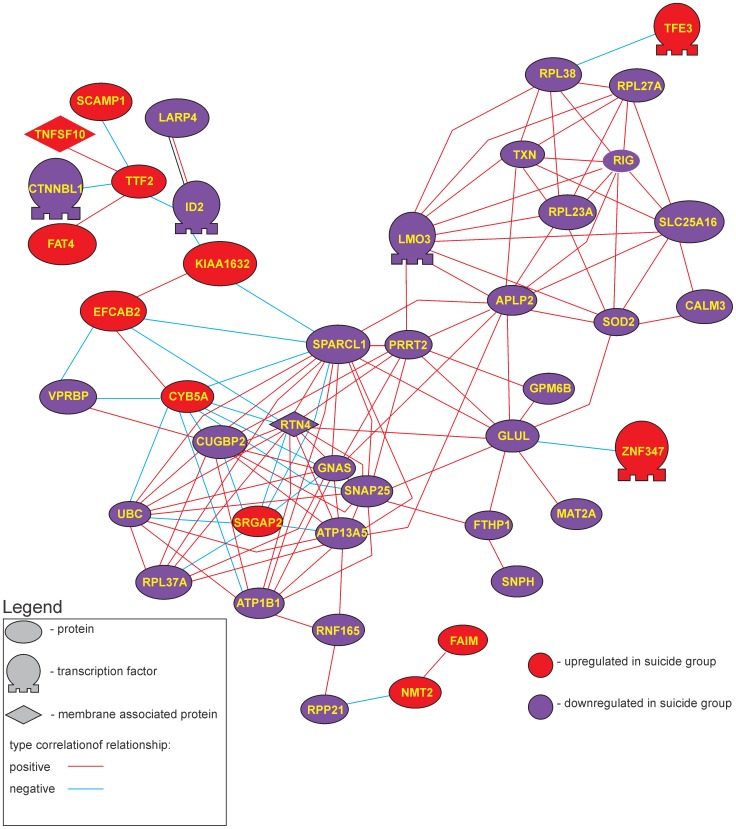
Coordinated gene expression is greatly reduced in suicide/MDD cohort. Pearson’s correlation-based gene network for differentially expressed genes for depressed suicide class. Red nodes represent genes up-regulated in depressed suicide class, blue nodes represent genes down-regulated in depressed suicide class, graphs - correlation of gene expression. Network contains 21 genes connected by 80 correlation relationships higher than 0.8.

As prior reports using QPCR analysis demonstrated a high degree of correlation of gene expression of GABA_A_ receptor sub-units in control brain relative to depressed suicide brain [1], [17], we analyzed whether a similar profile would be apparent in a much larger set of differentially expressed genes (i.e., in the 238 DEG in our data set). As doing analysis of all present genes in both cohorts independent of whether they were differentially expressed or not is not feasible/interpretable (due to high α error associated with so many comparisons), we limited the analysis to those genes that were differentially expressed, treating the two groups separately. In effect this analysis asks if the altered gene expression is accompanied by a loss of coordinated expression as well. Pearson’s correlation-based gene networks generated from the expression data (the 340 differentially expressed probe sets) revealed 45 genes with 134 relationships having a correlation coefficient *r* >0.8 (positive or negative) at *p*<0.01 (Figure 7). Examination of the same gene list in the depressed/suicide cohort showed far fewer such relationships; only 21 genes with 80 correlations were identified (Figure 8). GO analysis of these genes again showed the cellular processes involved in synaptic transmission and cell to cell adhesion were enriched in this list (see Table 6 and Table S2).

**Table 6:** Summary of GO analysis of DEGs found to be significantly correlated in control and MDD suicide subjects.

Correlated in control group		
GO Term	Gene count	*p*
translational elongation	5	1.1E−04
regulation of morphology	3	4.6E−03
regulation of development	7	5.8E−03
regulation of cell differentiation	6	6.9E−03
translation	5	8.8E−03
cellular homeostasis	33	1.2E−02
synaptic vesicle docking during exocytosis	2	1.2E−02
regulation of differentiation	3	1.3E−02
secretion by cell	4	1.4E−02
apoptosis	6	1.6E−02
**Correlated in suicide group**		
**GO Term**	**Gene count**	***p***
regulation of cellular component biogenesis	3	1.2E−02
regulation of cellular component organization	4	1.6E−02
regulation of synaptogenesis	2	2.1E−02
regulation of nervous system development	3	2.2E−02
regulation of synapse organization	2	2.5E−02
regulation of synapse structure and activity	2	2.9E−02
regulation of developmental process	4	4.5E−02
synaptic transmission	3	4.9E−02
regulation of developmental growth	2	5.5E−02
nitrogen compound biosynthetic process	3	5.7E−02

## Discussion

The present findings revealed that in the frontopolar cortex of depressed individuals that died by suicide, networks of gene products exist that appear to be dysregulated relative to the non-depressed cohort that died quickly of causes other than suicide. We used two types of analysis to provide insight into how the biology of the depressed suicide brain might be different from normal controls. The first, pathway analysis, provided the gene networks shown in Figure 5 which was based on the “reading” of the scientific literature by the software. The second set of networks (Figures 7–8) was created by an analysis of gene expression correlations in controls and in depressed individuals that died by suicide. These analyses indicated agreement in the biological processes that were implicated as being different in the depressed suicide brain. The potential biological processes that have been implicated for these networks are listed in Table 6 (and Table S3). Although the functional relevance of a few processes are obviously difficult to reconcile with known brain functions (myeloid leukocyte differentiation for example), the overwhelming majority are involved in regulatory or developmental process, synaptic communication and cell to cell interactions.

These conclusions are based on a computational analysis which relies on identifying functional relationships that have been established to various degrees of certainty in the biomedical literature. The analysis enables the effective access of 21 million PubMed abstracts and 61 full text journals that cover mammalian biology, a task that is obviously not possible by conventional reading of the scientific literature. Although this analysis, at face value appears useful and exciting, there is, simply by sheer number of possible interactions analyzed, the question of whether or not this analysis is valid. First, some proteins have many functions and will therefore link with many others. Enzymes may have many targets or substrates and so relationships that are reported by this analysis may not be valid within a certain cell type that does not express a target molecule. The analysis is only as good as the present day knowledge of the protein interactions that are reported. There is also no “quality control” for the validity of the data contained in the publications. These limitations make the GO analysis particularly important as it identifies the overall implications of the gene interactions that are being reported.

These caveats notwithstanding, our data/analysis is remarkably consistent in that each GO analysis generated similar functional groups, those involved in cell structure and communication. As well, we showed that the pathway analysis of a random selection of the same number of genes did not provide apparently biologically relevant networks. This conclusion is based on the pathway analysis of the 100 random probe set lists of the same size where we reconstructed relationship networks and then assessed number of proteins and relationships in these networks. A statistical analysis showed that random networks identified by the pathway analysis were significantly smaller and less connected than the networks generated from the experimentally derived DEG list. This indicates that the interrelations found through the pathway analysis likely represent true biological differences between controls and depressed suicide samples. The function of this network is implicated in cell to cell communication as many of the processes suggested are involved in cell adhesion, cell morphology and synapse formation. A similar approach, commonly referred to as “bootstrapping” is a standard statistical procedure in other studies [18] but to our knowledge has not been done before in an analysis of this kind.

The pathway analysis also identified five genes that appeared as “network hubs” with connectivity higher than 20: RAC1, CTNNB1, STAT3, EP300 and PTK2. These genes are of interest as they represent the central points in cellular machinery that have relationships with a variety of other proteins. For example, there are more than 1500 relationships currently known for each of the five aforementioned proteins. Importantly, each of these has an established role in nervous system processes, having been implicated in nervous system development, neuronal migration and differentiation, neuroprotection and neuronal plasticity (Table 3).

It is also interesting that 11 transcription factors were identified that regulate at least two genes from the initial network. Of these, 8 have well established roles in nervous system functioning. Specifically, SP1, SP3 and RELA are involved in neurite growth, myelination and neuron survival [19]–[22], and TCF4 is important for nervous system development, axon morphogenesis and oligodendrocyte differentiation [23]. FOXO3 [24] regulates neural progenitor stem cell proliferation as well as the induction of genomic death responses upon its’ translocation from the cytosol to the nucleus in response to excitotoxic stimuli [25]. MeCP2 is one of the central factors involved in gene regulation through differential CpG methylation in various tissues and organs, including the nervous system [26]. Finally, CREB1 and EGR1 have well established roles in synaptic plasticity, learning and memory [27]–[30].

We also found 7 genes that were under control of at least 2 transcription factors implicated in the direct relationship network in Figure 3. Of these, 5 have a well-established role in nervous system functioning. FAS is involved in neuronal development and degeneration [31], [32], CCND1 is important for neuronal cells proliferation [33], [34], and GFAP and ERBB2 are involved in glia and Schwann cell function, as well as synapse formation and maturation [35], [36]. Furthermore, VEGFA is involved in angiogenesis and is also implicated in development of amyotrophic lateral sclerosis (ALS) [37], [38]. How any of these factors contribute to the underlying depressive behavior is unclear, but the wide ranging effects on the expression of these transcription factors suggest broad disturbances in gene function.

### Networks Identified through Correlation Analyses

As we have previously shown in a much more limited analysis, gene expression in MDD/suicide brain seems to be much less coordinated than in control samples [8]. In the correlation analyses done here we found 43 correlated genes in the control (non-depressed) class, forming two distinct sub-networks with 134 correlated relationships. In the depressed/suicide condition, by contrast, only 21 proteins were significantly correlated to one another. The loss of coordinated expression seemed to be more profound among genes that were up-regulated in MDD/suicide group. Specifically, in the depressed/suicide PC networks, 12 DEG’s were up-regulated in the depressed/suicide class and 33 were down-regulated (approx. 1∶3). In MDD/suicide PC network, in contrast, only 2 DEG’s were up-regulated and 19 were down-regulated (approx. 1∶10). This suggests that the down-regulation is “concerted”, whereas genes that were up-regulated in expression appear to do so in a more “random” manner. Overall this analysis suggests that in depressed/suicide individuals there is a wide ranging loss of organized expression among genes that are important for determining the wiring neural networks.

A number of genes were down-regulated in depressed/suicide tissue, but were not present in the network (Figure 5) that nonetheless would be predicted to have wide ranging effects on synaptic function. One of these, GNAS (stimulatory alpha subunit of G protein), is involved in many neurotransmitter signaling cascades, including 5-HT and dopamine receptors. Another is RTN4 or neurite outgrowth inhibitor, a regulator of apoptosis and was implicated in neurodevelopmental processes [39]–[41]. As well, the expression of synaptosomal-associated protein 25 (SNAP25), which is involved in synaptic vesicle membrane docking and fusion and regulation of neurotransmitter release was reduced. The down-regulation of this protein has also been implicated in several psychiatric disorders [42]. In addition, SPARCL1 or hevin is a putative extracellular matrix glycoprotein that binds calcium and plays an important role in the developing nervous system [43]. Finally, GLUL or glutamate-ammonia ligase clears L-glutamate, the major neurotransmitter in the central nervous system, from neuronal synapses [44]. Overall these changes implicate a profound disturbance in excitatory amino transmission irrespective of any other changes in gene expression within the networks identified. The fact that they appear to be altered in expression but are not represented in the largest network identified (Figure 5) suggests a wider disturbance in the gene expression than was identified by pathway analysis.

One of the limitations of this study is the small n of both the control and sample groups. This was deliberate as we choose to analyze a small number of well-matched samples (similar age RIN sex etc) rather than a larger more variable cohort. For the correlation analysis, where hundreds of correlations were compared, the α error may be potentially high, however, this ought to be comparable in both groups. Thus our results showing many fewer correlations in depressed/suicide cannot be attributed to the number of genes compared. We should also note that some genes that we and others have found to be down-regulated (BDNF, GABRA1 [1], [45] ) were not found in these analyses (although they were near the cut-off used here). This is likely attributed to the fact that although microarray analysis is very reproducible is not very sensitive in detecting small changes in gene expression. This fact can be observed in Figure 2 where a 1.3 fold change in expression detected on the microarray correlated to more than 2 cycles of change in the QPCR data. Thus the differences reported here are likely the largest differences in gene expression between these two groups. Finally, our cohort is also confounded by the fact that the samples come from those who also committed suicide. Thus our findings may be relevant to suicidality and/or depression. Recent studies by Turecki and others have shown that there may be gene expression patterns that are associated with suicidality [6], [46] albeit with some overlap in those associated MDD. This overlap is a significant confound in “teasing out” the gene expression that is unique to these two psychiatric states. Studies of gene expression in those with MDD who did not die from suicide found two highly dysregulated genes including stresscopin, a neuropeptide involved in stress responses and Forkhead box D3 (FOXD3), a transcription factor as well as factors related to synapse formation [47], [48]. The finding of another Forkhead box transcription factor is similar to the data reported here where we found FOXO3 and many factors related to synaptogenesis/maintenance. This suggests that our data speak more to depressive syndrome rather suicidality.

In summary, the present findings indicate that among depressed individuals that died by suicide, profound alterations exist in the expression of genes that control synaptic function, cell adhesion and cytoarchitecture. They also extend and support our observation [8], [17] that coordinated gene expression is apparently disturbed in the MDD/suicide samples in comparison to normal controls. Interestingly, we also found that in mice acute and chronic stressors can also alter coordinated gene expression of the GABA_A_ receptor gene cassette [49]. As stressful events may be a precipitating factor in the development of MDD, it might be important to identify the biochemical and/or epigenetic processes that disturb normal gene expression. These data also provide a number of new targets for interventions that could help treat MDD.

## Supporting Information

Table S1 **GO Analysis of Differentially Expressed Gene List (p<0.01 FC >1.3).**
(DOCX)

Table S2 **GO analysis of DEGS from pathway analysis (largest network).**
(DOCX)

Table S3 **GO analysis of genes correlated in suicide group.**
(DOCX)
