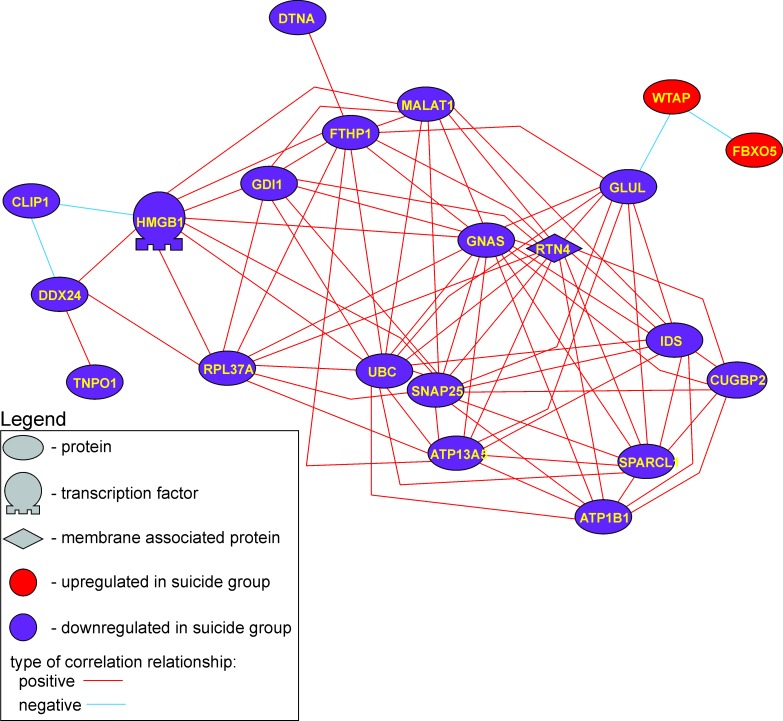# Correction: Molecular Pathway Reconstruction and Analysis of Disturbed Gene Expression in Depressed Individuals Who Died by Suicide

**DOI:** 10.1371/annotation/a023885b-a395-4d6c-9f7c-fe255675f171

**Published:** 2013-02-27

**Authors:** Vladimir Zhurov, John D. H. Stead, Zul Merali, Miklos Palkovits, Gabor Faludi, Caroline Schild-Poulter, Hymie Anisman, Michael O. Poulter

Figure 8 was incorrectly made a duplicate of Figure 7. The correct Figure 8 is available here: 

**Figure:**