# Supplementary material for: Molecular Pathway Reconstruction and Analysis of Disturbed Gene Expression in Depressed Individuals Who Died by Suicide
Source: PLoS One. 2012 Oct 22;7(10):e47581. doi: 10.1371/journal.pone.0047581 (PMC3478292; doi:10.1371/journal.pone.0047581)
Supplement: Table S1 — GO Analysis of Differentially Expressed Gene List (p<0.01 FC >1.3). (DOCX) [file pone.0047581.s001.docx]

| Table S1 |  |
| --- | --- |
|  |  |

| **Cellular homeostasis and signaling** | |
| --- | --- |
| ACN9 | ACN9 homolog (S. cerevisiae) |
| AFF3 | AF4/FMR2 family, member 3 |
| APLP2 | amyloid beta (A4) precursor-like protein 2 |
| ARG2 | arginase, type II |
| ARID1B | AT rich interactive domain 1B (SWI1-like) |
| ATP13A5 | ATPase type 13A5 |
| ATP1B1 | ATPase, Na+/K+ transporting, beta 1 polypeptide |
| ATP6V0A1 | ATPase, H+ transporting, lysosomal V0 subunit a1 |
| ATP6V0E1 | ATPase, H+ transporting, lysosomal 9kDa, V0 subunit e1 |
| BMPR1B | bone morphogenetic protein receptor, type IB |
| C1D | C1D nuclear receptor co-repressor |
| CACNB2 | calcium channel, voltage-dependent, beta 2 subunit |
| CACNB4 | calcium channel, voltage-dependent, beta 4 subunit |
| CACNG2 | calcium channel, voltage-dependent, gamma subunit 2 |
| CALM3 | calmodulin 3 (phosphorylase kinase, delta) |
| CALR | Calreticulin |
| CAMK2G | calcium/calmodulin-dependent protein kinase II gamma |
| CAPZA2 | capping protein (actin filament) muscle Z-line, alpha 2 |
| CCDC76 | coiled-coil domain containing 76 |
| CCNC | cyclin C |
| CDC14B | CDC14 cell division cycle 14 homolog B (S. cerevisiae) |
| CLCN4 | chloride channel 4 |
| CLIP1 | CAP-GLY domain containing linker protein 1 |
| CLTA | clathrin, light chain (Lca) |
| CPSF6 | cleavage and polyadenylation specific factor 6, 68kDa |
| CREM | cAMP responsive element modulator |
| CSNK2A2 | casein kinase 2, alpha prime polypeptide |
| CTNNB1 | catenin (cadherin-associated protein), beta 1, 88kDa |
| CTNNBL1 | catenin, beta like 1 |
| CUL4A | cullin 4A |
| CYB5A | cytochrome b5 type A (microsomal) |
| DAD1 | defender against cell death 1 |
| DBNL | drebrin-like |
| DCLK1 | doublecortin-like kinase 1 |
| DDX24 | DEAD (Asp-Glu-Ala-Asp) box polypeptide 24 |
| DIDO1 | death inducer-obliterator 1 |
| DTNA | dystrobrevin, alpha |
| DUSP28 | dual specificity phosphatase 28 |
| EEA1 | early endosome antigen 1 |
| EIF2A | eukaryotic translation initiation factor 2A, 65kDa |
| EIF5B | eukaryotic translation initiation factor 5B |
| EME1 | essential meiotic endonuclease 1 homolog 1 (S. pombe) |
| ENPP6 | ectonucleotide pyrophosphatase/phosphodiesterase 6 |
| EP300 | E1A binding protein p300 |
| FAIM | Fas apoptotic inhibitory molecule |
| FASN | fatty acid synthase |
| FAT4 | FAT tumor suppressor homolog 4 (Drosophila) |
| FBL | fibrillarin |
| FBXO31 | F-box protein 31 |
| FBXO5 | F-box protein 5 |
| FLII | flightless I homolog (Drosophila) |
| FOXN3 | forkhead box N3 |
| FUT6 | fucosyltransferase 6 (alpha (1,3) fucosyltransferase) |
| GABBR2 | gamma-aminobutyric  acid (GABA) B receptor, 2 |
| GALNT7 | UDP-N-acetyl-alpha-D-galactosamine:polypeptide N-acetylgalactosaminyltransferase 7 (GalNAc-T7) |
| GLRX3 | glutaredoxin 3 |
| GLS | glutaminase |
| GLUL | glutamate-ammonia ligase (glutamine synthetase) |
| GNAS | GNAS complex locus |
| GPM6B | glycoprotein M6B |
| GTF2H2 | general transcription factor IIH |
| HDAC7 | histone deacetylase 7 |
| HMGB1 | high-mobility group box 1; high-mobility group box 1-like 10 |
| HRH3 | histamine receptor H3 |
| ID2 | inhibitor of DNA binding 2, dominant negative helix-loop-helix protein |
| IFT20 | intraflagellar transport 20 homolog (Chlamydomonas) |
| IL6R | interleukin 6 receptor |
| IPO11 | importin 11 |
| ITPR1 | inositol 1,4,5-triphosphate receptor, type 1 |
| KDR | kinase insert domain receptor (a type III receptor tyrosine kinase) |
| KIF1B | kinesin family member 1B |
| LEPR | leptin receptor |
| LMO3 | LIM domain only 3 (rhombotin-like 2) |
| LNPEP | leucyl/cystinyl aminopeptidase |
| LRRFIP1 | leucine rich repeat (in FLII) interacting protein 1 |
| MAT2A | methionine adenosyltransferase II, alpha |
| MEF2D | myocyte enhancer factor 2D |
| MRPL30 | mitochondrial ribosomal protein L30 |
| MRPL52 | mitochondrial ribosomal protein L52 |
| MRS2 | MRS2 magnesium homeostasis factor homolog (S. cerevisiae) |
| MSRA | methionine sulfoxide reductase A |
| NCOR2 | nuclear receptor co-repressor 2 |
| NMT2 | N-myristoyltransferase 2 |
| NR2C1 | nuclear receptor subfamily 2, group C, member 1 |
| NRCAM | neuronal cell adhesion molecule |
| NRP2 | neuropilin 2 |
| PDP2 | pyruvate dehyrogenase phosphatase catalytic subunit 2 |
| PDXDC2 | pyridoxal-dependent decarboxylase domain containing 2 |
| PIGF | phosphatidylinositol glycan anchor biosynthesis, class F |
| PLAGL1 | pleiomorphic adenoma gene-like 1 |
| PPP1R15A | protein phosphatase 1, regulatory (inhibitor) subunit 15A |
| PPP3CA | protein phosphatase 3 (formerly 2B), catalytic subunit, alpha isoform |
| PRDX4 | peroxiredoxin 4 |
| PTK2 | PTK2 protein tyrosine kinase 2 |
| RAB7A | RAB7A, member RAS oncogene family |
| RAC1 | ras-related C3 botulinum toxin substrate 1 (rho family, small GTP binding protein Rac1) |
| RBBP6 | retinoblastoma binding protein 6 |
| RBMS1 | RNA binding motif, single stranded interacting protein 1 |
| REC8 | REC8 homolog (yeast) |
| RERE | arginine-glutamic acid dipeptide (RE) repeats |
| RGMB | RGM domain family, member B |
| RIT2 | Ras-like without CAAX 2 |
| RPGR | retinitis pigmentosa GTPase regulator |
| RPL13A | ribosomal protein L13a pseudogene 7 |
| RPL23A | ribosomal protein L23a pseudogene |
| RPL27A | ribosomal protein L27a |
| RPL37A | ribosomal protein L37a |
| RPL38 | ribosomal protein L38 |
| RPP21 | ribonuclease P/MRP 21kDa subunit |
| RPS11 | ribosomal protein S11 pseudogene 5; ribosomal protein S11 |
| RTN4 | reticulon 4 |
| SCAMP1 | secretory carrier membrane protein 1 |
| SEMA6B | sema domain, transmembrane domain (TM), and cytoplasmic domain, (semaphorin) 6B |
| SERBP1 | SERPINE1 mRNA binding protein 1 |
| SLC25A16 | solute carrier family 25 (mitochondrial carrier; Graves disease autoantigen), member 16 |
| SLC30A5 | solute carrier family 30 (zinc transporter), member 5 |
| SLC35A3 | solute carrier family 35 (UDP-N-acetylglucosamine (UDP-GlcNAc) transporter), member A3 |
| SNAP25 | synaptosomal-associated protein, 25kDa |
| SNPH | syntaphilin; RAD21-like 1 (S. pombe) |
| SNX2 | sorting nexin 2 |
| SOD2 | superoxide dismutase 2, mitochondrial |
| SPTBN1 | spectrin, beta, non-erythrocytic 1 |
| SQLE | squalene epoxidase |
| SRP72 | signal recognition particle 72kDa |
| STAM2 | signal transducing adaptor molecule (SH3 domain and ITAM motif) 2 |
| STAT3 | signal transducer and activator of transcription 3 (acute-phase response factor) |
| SYPL1 | synaptophysin-like 1 |
| TACC2 | transforming, acidic coiled-coil containing protein 2 |
| TAF1A | TATA box binding protein (TBP)-associated factor, RNA polymerase I, A, 48kDa |
| TCP1 | hypothetical gene supported by BC000665; t-complex 1 |
| TFE3 | transcription factor binding to IGHM enhancer 3 |
| THTPA | thiamine triphosphatase |
| TIMP2 | TIMP metallopeptidase inhibitor 2 |
| TINF2 | TERF1 (TRF1)-interacting nuclear factor 2 |
| TNFSF10 | tumor necrosis factor (ligand) superfamily, member 10 |
| TNPO1 | transportin 1 |
| TRPC1 | transient receptor potential cation channel, subfamily C, member 1 |
| TSPYL1 | TSPY-like 1 |
| TTC9B | tetratricopeptide repeat domain 9B |
| TTF2 | transcription termination factor, RNA polymerase II |
| TUSC3 | tumor suppressor candidate 3 |
| TXN | thioredoxin |
| UBC | ubiquitin C |
| USP2 | ubiquitin specific peptidase 2 |
| USP38 | ubiquitin specific peptidase 38 |
| VCAM1 | vascular cell adhesion molecule 1 |
| VPRBP | Vpr (HIV-1) binding protein |
| VPS13A | vacuolar protein sorting 13 homolog A (S. cerevisiae) |
| WDR12 | WD repeat domain 12 |
| WTAP | Wilms tumor 1 associated protein |
| XPO7 | exportin 7 |
| XRCC5 | X-ray repair complementing defective repair in Chinese hamster cells 5 (double-strand-break rejoining) |
| YAF2 | YY1 associated factor 2 |
| ZNF131 | zinc finger protein 131 |
| ZNF182 | zinc finger protein 182 |
| ZNF286A | zinc finger protein 286A |
| ZNF347 | zinc finger protein 347 |
| ZNF71 | zinc finger protein 71 |
|  |  |
| **synaptic transmission** | |
| CACNB2 | calcium channel, voltage-dependent, beta 2 subunit |
| CACNB4 | calcium channel, voltage-dependent, beta 4 subunit |
| CTNNB1 | catenin (cadherin-associated protein), beta 1, 88kDa |
| DTNA | dystrobrevin, alpha |
| GABBR2 | gamma-aminobutyric acid (GABA) B receptor, 2 |
| HRH3 | histamine receptor H3 |
| KIF1B | kinesin family member 1B |
| PPP3CA | protein phosphatase 3 (formerly 2B), catalytic subunit, alpha isoform |
| PTK2 | PTK2 protein tyrosine kinase 2 |
| RIT2 | Ras-like without CAAX 2 |
| SNAP25 | synaptosomal-associated protein, 25kDa |
| SNPH | syntaphilin; RAD21-like 1 (S. pombe) |
| SYPL1 | synaptophysin-like 1 |
| UBC | ubiquitin C |
|  |  |
| **cellular localization** | |
| CALR | calreticulin |
| CAMK2G | calcium/calmodulin-dependent protein kinase II gamma |
| CLTA | clathrin, light chain (Lca) |
| CTNNB1 | catenin (cadherin-associated protein), beta 1, 88kDa |
| DCLK1 | doublecortin-like kinase 1 |
| EEA1 | early endosome antigen 1 |
| GNAS | GNAS complex locus |
| HRH3 | histamine receptor H3 |
| IPO11 | importin 11 |
| KIF1B | kinesin family member 1B |
| PPP3CA | protein phosphatase 3 (formerly 2B), catalytic subunit, alpha isoform |
| PTK2 | PTK2 protein tyrosine kinase 2 |
| RAC1 | ras-related C3 botulinum toxin substrate 1 (rho family, small GTP binding protein Rac1) |
| RERE | arginine-glutamic acid dipeptide (RE) repeats |
| RPGR | retinitis pigmentosa GTPase regulator |
| SCAMP1 | secretory carrier membrane protein 1 |
| SNAP25 | synaptosomal-associated protein, 25kDa |
| SNPH | syntaphilin; RAD21-like 1 (S. pombe) |
| SNX2 | sorting nexin 2 |
| SPTBN1 | spectrin, beta, non-erythrocytic 1 |
| SRP72 | signal recognition particle 72kDa |
| STAM2 | signal transducing adaptor molecule (SH3 domain and ITAM motif) 2 |
| TACC2 | transforming, acidic coiled-coil containing protein 2 |
| TINF2 | TERF1 (TRF1)-interacting nuclear factor 2 |
| TNPO1 | transportin 1 |
| VPS13A | vacuolar protein sorting 13 homolog A (S. cerevisiae) |
| XPO7 | exportin 7 |
|  |  |
| **transmission of nerve impulse** | |
| CACNB2 | calcium channel, voltage-dependent, beta 2 subunit |
| CACNB4 | calcium channel, voltage-dependent, beta 4 subunit |
| CACNG2 | calcium channel, voltage-dependent, gamma subunit 2 |
| CTNNB1 | catenin (cadherin-associated protein), beta 1, 88kDa |
| DTNA | dystrobrevin, alpha |
| GABBR2 | gamma-aminobutyric acid (GABA) B receptor, 2 |
| HRH3 | histamine receptor H3 |
| KIF1B | kinesin family member 1B |
| PPP3CA | protein phosphatase 3 (formerly 2B), catalytic subunit, alpha isoform |
| PTK2 | PTK2 protein tyrosine kinase 2 |
| RIT2 | Ras-like without CAAX 2 |
| SNAP25 | synaptosomal-associated protein, 25kDa |
| SNPH | syntaphilin; RAD21-like 1 (S. pombe) |
| SYPL1 | synaptophysin-like 1 |
| UBC | ubiquitin C |
|  |  |
| **localization** | |
| ABCA5 | ATP-binding cassette, sub-family A (ABC1), member 5 |
| ATP13A5 | ATPase type 13A5 |
| ATP1B1 | ATPase, Na+/K+ transporting, beta 1 polypeptide |
| ATP6V0A1 | ATPase, H+ transporting, lysosomal V0 subunit a1 |
| ATP6V0E1 | ATPase, H+ transporting, lysosomal 9kDa, V0 subunit e1 |
| CACNB2 | calcium channel, voltage-dependent, beta 2 subunit |
| CACNB4 | calcium channel, voltage-dependent, beta 4 subunit |
| CACNG2 | calcium channel, voltage-dependent, gamma subunit 2 |
| CALR | calreticulin |
| CAMK2G | calcium/calmodulin-dependent protein kinase II gamma |
| CHMP2B | chromatin modifying protein 2B |
| CLCN4 | chloride channel 4 |
| CLTA | clathrin, light chain (Lca) |
| CTNNB1 | catenin (cadherin-associated protein), beta 1, 88kDa |
| CYB5A | cytochrome b5 type A (microsomal) |
| DBNL | drebrin-like |
| DCLK1 | doublecortin-like kinase 1 |
| EEA1 | early endosome antigen 1 |
| FMR1 | fragile X mental retardation 1 |
| GDI1 | GDP dissociation inhibitor 1 |
| GNAS | GNAS complex locus |
| GTF2H2 | general transcription factor IIH |
| HRH3 | histamine receptor H3 |
| IL6R | interleukin 6 receptor |
| IPO11 | importin 11 |
| ITPR1 | inositol 1,4,5-triphosphate receptor, type 1 |
| KDR | kinase insert domain receptor (a type III receptor tyrosine kinase) |
| KIF1B | kinesin family member 1B |
| MRS2 | MRS2 magnesium homeostasis factor homolog (S. cerevisiae) |
| NRCAM | neuronal cell adhesion molecule |
| NRP2 | neuropilin 2 |
| PITPNC1 | phosphatidylinositol transfer protein, cytoplasmic 1 |
| PPP3CA | protein phosphatase 3 (formerly 2B), catalytic subunit, alpha isoform |
| PTK2 | PTK2 protein tyrosine kinase 2 |
| RAB7A | RAB7A, member RAS oncogene family |
| RAC1 | ras-related C3 botulinum toxin substrate 1 (rho family, small GTP binding protein Rac1) |
| RALBP1 | hypothetical LOC100129773; ralA binding protein 1 |
| RERE | arginine-glutamic acid dipeptide (RE) repeats |
| RPGR | retinitis pigmentosa GTPase regulator |
| SCAMP1 | secretory carrier membrane protein 1 |
| SLC25A16 | solute carrier family 25 (mitochondrial carrier; Graves disease autoantigen), member 16 |
| SLC30A5 | solute carrier family 30 (zinc transporter), member 5 |
| SLC35A3 | solute carrier family 35 (UDP-N-acetylglucosamine (UDP-GlcNAc) transporter), member A3 |
| SNAP25 | synaptosomal-associated protein, 25kDa |
| SNPH | syntaphilin; RAD21-like 1 (S. pombe) |
| SNX2 | sorting nexin 2 |
| SPTBN1 | spectrin, beta, non-erythrocytic 1 |
| SRP72 | signal recognition particle 72kDa |
| STAM2 | signal transducing adaptor molecule (SH3 domain and ITAM motif) 2 |
| SYPL1 | synaptophysin-like 1 |
| TACC2 | transforming, acidic coiled-coil containing protein 2 |
| TINF2 | TERF1 (TRF1)-interacting nuclear factor 2 |
| TMED4 | transmembrane emp24 protein transport domain containing 4 |
| TNPO1 | transportin 1 |
| TRPC1 | transient receptor potential cation channel, subfamily C, member 1 |
| TXN | thioredoxin |
| VCAM1 | vascular cell adhesion molecule 1 |
| VPS13A | vacuolar protein sorting 13 homolog A (S. cerevisiae) |
| VTA1 | Vps20-associated 1 homolog (S. cerevisiae) |
| XPO7 | exportin 7 |
|  |  |
| **homeostatic process** | |
| APLP2 | amyloid beta (A4) precursor-like protein 2 |
| ATP6V0E1 | ATPase, H+ transporting, lysosomal 9kDa, V0 subunit e1 |
| CACNB4 | calcium channel, voltage-dependent, beta 4 subunit |
| CACNG2 | calcium channel, voltage-dependent, gamma subunit 2 |
| CALR | calreticulin |
| CTNNB1 | catenin (cadherin-associated protein), beta 1, 88kDa |
| EP300 | E1A binding protein p300 |
| GLRX3 | glutaredoxin 3 |
| HRH3 | histamine receptor H3 |
| ID2 | inhibitor of DNA binding 2, dominant negative helix-loop-helix protein |
| ITPR1 | inositol 1,4,5-triphosphate receptor, type 1 |
| KDR | kinase insert domain receptor (a type III receptor tyrosine kinase) |
| PPP3CA | protein phosphatase 3 (formerly 2B), catalytic subunit, alpha isoform |
| PRDX4 | peroxiredoxin 4 |
| RAC1 | ras-related C3 botulinum toxin substrate 1 (rho family, small GTP binding protein Rac1) |
| SLC30A5 | solute carrier family 30 (zinc transporter), member 5 |
| SOD2 | superoxide dismutase 2, mitochondrial |
| STAT3 | signal transducer and activator of transcription 3 (acute-phase response factor) |
| TINF2 | TERF1 (TRF1)-interacting nuclear factor 2 |
| TRPC1 | transient receptor potential cation channel, subfamily C, member 1 |
| TXN | thioredoxin |
| XRCC5 | X-ray repair complementing defective repair in Chinese hamster cells 5 (double-strand-break rejoining) |
|  |  |
| **establishment of localization** | |
| ABCA5 | ATP-binding cassette, sub-family A (ABC1), member 5 |
| ATP13A5 | ATPase type 13A5 |
| ATP1B1 | ATPase, Na+/K+ transporting, beta 1 polypeptide |
| ATP6V0A1 | ATPase, H+ transporting, lysosomal V0 subunit a1 |
| ATP6V0E1 | ATPase, H+ transporting, lysosomal 9kDa, V0 subunit e1 |
| CACNB2 | calcium channel, voltage-dependent, beta 2 subunit |
| CACNB4 | calcium channel, voltage-dependent, beta 4 subunit |
| CACNG2 | calcium channel, voltage-dependent, gamma subunit 2 |
| CALR | calreticulin |
| CAMK2G | calcium/calmodulin-dependent protein kinase II gamma |
| CHMP2B | chromatin modifying protein 2B |
| CLCN4 | chloride channel 4 |
| CLTA | clathrin, light chain (Lca) |
| CTNNB1 | catenin (cadherin-associated protein), beta 1, 88kDa |
| CYB5A | cytochrome b5 type A (microsomal) |
| DBNL | drebrin-like |
| DCLK1 | doublecortin-like kinase 1 |
| EEA1 | early endosome antigen 1 |
| FMR1 | fragile X mental retardation 1 |
| GDI1 | GDP dissociation inhibitor 1 |
| GNAS | GNAS complex locus |
| GTF2H2 | general transcription factor IIH |
| HRH3 | histamine receptor H3 |
| IPO11 | importin 11 |
| ITPR1 | inositol 1,4,5-triphosphate receptor, type 1 |
| KIF1B | kinesin family member 1B |
| MRS2 | MRS2 magnesium homeostasis factor homolog (S. cerevisiae) |
| PITPNC1 | phosphatidylinositol transfer protein, cytoplasmic 1 |
| PPP3CA | protein phosphatase 3 (formerly 2B), catalytic subunit, alpha isoform |
| PTK2 | PTK2 protein tyrosine kinase 2 |
| RAB7A | RAB7A, member RAS oncogene family |
| RAC1 | ras-related C3 botulinum toxin substrate 1 (rho family, small GTP binding protein Rac1) |
| RALBP1 | hypothetical LOC100129773; ralA binding protein 1 |
| RERE | arginine-glutamic acid dipeptide (RE) repeats |
| RPGR | retinitis pigmentosa GTPase regulator |
| SCAMP1 | secretory carrier membrane protein 1 |
| SLC25A16 | solute carrier family 25 (mitochondrial carrier; Graves disease autoantigen), member 16 |
| SLC30A5 | solute carrier family 30 (zinc transporter), member 5 |
| SLC35A3 | solute carrier family 35 (UDP-N-acetylglucosamine (UDP-GlcNAc) transporter), member A3 |
| SNAP25 | synaptosomal-associated protein, 25kDa |
| SNPH | syntaphilin; RAD21-like 1 (S. pombe) |
| SNX2 | sorting nexin 2 |
| SPTBN1 | spectrin, beta, non-erythrocytic 1 |
| SRP72 | signal recognition particle 72kDa |
| STAM2 | signal transducing adaptor molecule (SH3 domain and ITAM motif) 2 |
| SYPL1 | synaptophysin-like 1 |
| TMED4 | transmembrane emp24 protein transport domain containing 4 |
| TNPO1 | transportin 1 |
| TRPC1 | transient receptor potential cation channel, subfamily C, member 1 |
| TXN | thioredoxin |
| VPS13A | vacuolar protein sorting 13 homolog A (S. cerevisiae) |
| VTA1 | Vps20-associated 1 homolog (S. cerevisiae) |
| XPO7 | exportin 7 |
|  |  |
| **transport** |  |
| ABCA5 | ATP-binding cassette, sub-family A (ABC1), member 5 |
| ATP13A5 | ATPase type 13A5 |
| ATP1B1 | ATPase, Na+/K+ transporting, beta 1 polypeptide |
| ATP6V0A1 | ATPase, H+ transporting, lysosomal V0 subunit a1 |
| ATP6V0E1 | ATPase, H+ transporting, lysosomal 9kDa, V0 subunit e1 |
| CACNB2 | calcium channel, voltage-dependent, beta 2 subunit |
| CACNB4 | calcium channel, voltage-dependent, beta 4 subunit |
| CACNG2 | calcium channel, voltage-dependent, gamma subunit 2 |
| CALR | calreticulin |
| CAMK2G | calcium/calmodulin-dependent protein kinase II gamma |
| CHMP2B | chromatin modifying protein 2B |
| CLCN4 | chloride channel 4 |
| CLTA | clathrin, light chain (Lca) |
| CTNNB1 | catenin (cadherin-associated protein), beta 1, 88kDa |
| CYB5A | cytochrome b5 type A (microsomal) |
| DBNL | drebrin-like |
| DCLK1 | doublecortin-like kinase 1 |
| EEA1 | early endosome antigen 1 |
| FMR1 | fragile X mental retardation 1 |
| GDI1 | GDP dissociation inhibitor 1 |
| GNAS | GNAS complex locus |
| GTF2H2 | general transcription factor IIH |
| HRH3 | histamine receptor H3 |
| IPO11 | importin 11 |
| ITPR1 | inositol 1,4,5-triphosphate receptor, type 1 |
| KIF1B | kinesin family member 1B |
| MRS2 | MRS2 magnesium homeostasis factor homolog (S. cerevisiae) |
| PITPNC1 | phosphatidylinositol transfer protein, cytoplasmic 1 |
| PPP3CA | protein phosphatase 3 (formerly 2B), catalytic subunit, alpha isoform |
| RAB7A | RAB7A, member RAS oncogene family |
| RAC1 | ras-related C3 botulinum toxin substrate 1 (rho family, small GTP binding protein Rac1) |
| RALBP1 | hypothetical LOC100129773; ralA binding protein 1 |
| RERE | arginine-glutamic acid dipeptide (RE) repeats |
| RPGR | retinitis pigmentosa GTPase regulator |
| SCAMP1 | secretory carrier membrane protein 1 |
| SLC25A16 | solute carrier family 25 (mitochondrial carrier; Graves disease autoantigen), member 16 |
| SLC30A5 | solute carrier family 30 (zinc transporter), member 5 |
| SLC35A3 | solute carrier family 35 (UDP-N-acetylglucosamine (UDP-GlcNAc) transporter), member A3 |
| SNAP25 | synaptosomal-associated protein, 25kDa |
| SNPH | syntaphilin; RAD21-like 1 (S. pombe) |
| SNX2 | sorting nexin 2 |
| SPTBN1 | spectrin, beta, non-erythrocytic 1 |
| SRP72 | signal recognition particle 72kDa |
| STAM2 | signal transducing adaptor molecule (SH3 domain and ITAM motif) 2 |
| SYPL1 | synaptophysin-like 1 |
| TMED4 | transmembrane emp24 protein transport domain containing 4 |
| TNPO1 | transportin 1 |
| TRPC1 | transient receptor potential cation channel, subfamily C, member 1 |
| TXN | thioredoxin |
| VPS13A | vacuolar protein sorting 13 homolog A (S. cerevisiae) |
| VTA1 | Vps20-associated 1 homolog (S. cerevisiae) |
| XPO7 | exportin 7 |
|  |  |
| **establishment of localization in cell** | |
| CALR | calreticulin |
| CAMK2G | calcium/calmodulin-dependent protein kinase II gamma |
| CLTA | clathrin, light chain (Lca) |
| DCLK1 | doublecortin-like kinase 1 |
| EEA1 | early endosome antigen 1 |
| GNAS | GNAS complex locus |
| HRH3 | histamine receptor H3 |
| IPO11 | importin 11 |
| KIF1B | kinesin family member 1B |
| PPP3CA | protein phosphatase 3 (formerly 2B), catalytic subunit, alpha isoform |
| PTK2 | PTK2 protein tyrosine kinase 2 |
| RERE | arginine-glutamic acid dipeptide (RE) repeats |
| RPGR | retinitis pigmentosa GTPase regulator |
| SCAMP1 | secretory carrier membrane protein 1 |
| SNAP25 | synaptosomal-associated protein, 25kDa |
| SNPH | syntaphilin; RAD21-like 1 (S. pombe) |
| SNX2 | sorting nexin 2 |
| SPTBN1 | spectrin, beta, non-erythrocytic 1 |
| SRP72 | signal recognition particle 72kDa |
| STAM2 | signal transducing adaptor molecule (SH3 domain and ITAM motif) 2 |
| TNPO1 | transportin 1 |
| VPS13A | vacuolar protein sorting 13 homolog A (S. cerevisiae) |
| XPO7 | exportin 7 |
|  |  |
| **cell-cell signaling** | |
| CACNB2 | calcium channel, voltage-dependent, beta 2 subunit |
| CACNB4 | calcium channel, voltage-dependent, beta 4 subunit |
| CAMK2G | calcium/calmodulin-dependent protein kinase II gamma |
| CTNNB1 | catenin (cadherin-associated protein), beta 1, 88kDa |
| DTNA | dystrobrevin, alpha |
| GABBR2 | gamma-aminobutyric acid (GABA) B receptor, 2 |
| HRH3 | histamine receptor H3 |
| KIF1B | kinesin family member 1B |
| LNPEP | leucyl/cystinyl aminopeptidase |
| PPP3CA | protein phosphatase 3 (formerly 2B), catalytic subunit, alpha isoform |
| PTK2 | PTK2 protein tyrosine kinase 2 |
| RIT2 | Ras-like without CAAX 2 |
| SNAP25 | synaptosomal-associated protein, 25kDa |
| SNPH | syntaphilin; RAD21-like 1 (S. pombe) |
| SYPL1 | synaptophysin-like 1 |
| TNFSF10 | tumor necrosis factor (ligand) superfamily, member 10 |
| TXN | thioredoxin |
| UBC | ubiquitin C |

| **Supplemental** | **Table 2** |
| --- | --- |

**GO ANALYSIS OF DEGS FROM PATHWAY ANALYSIS (largest network)**

| **REGULATION OF CELL DIFFERENTIATION** | |
| --- | --- |
| CALR | calreticulin |
| CTNNB1 | catenin (cadherin-associated protein), beta 1, 88kDa |
| HDAC7 | histone deacetylase 7 |
| ID2 | inhibitor of DNA binding 2, dominant negative helix-loop-helix protein |
| IL6R | interleukin 6 receptor |
| NRCAM | neuronal cell adhesion molecule |
| PTK2 | PTK2 protein tyrosine kinase 2 |
| RTN4 | reticulon 4 |
| SOD2 | superoxide dismutase 2, mitochondrial |
| TFE3 | transcription factor binding to IGHM enhancer 3 |
| TIMP2 | TIMP metallopeptidase inhibitor 2 |
| XRCC5 | X-ray repair complementing defective repair in Chinese hamster cells 5 (double-strand-break rejoining) |
|  |  |
| **REGULATION OF DEVELOPMENTAL PROCESS** | |
| CALR | calreticulin |
| CTNNB1 | catenin (cadherin-associated protein), beta 1, 88kDa |
| HDAC7 | histone deacetylase 7 |
| ID2 | inhibitor of DNA binding 2, dominant negative helix-loop-helix protein |
| IL6R | interleukin 6 receptor |
| KDR | kinase insert domain receptor (a type III receptor tyrosine kinase) |
| NRCAM | neuronal cell adhesion molecule |
| PTK2 | PTK2 protein tyrosine kinase 2 |
| RTN4 | reticulon 4 |
| SOD2 | superoxide dismutase 2, mitochondrial |
| TFE3 | transcription factor binding to IGHM enhancer 3 |
| TIMP2 | TIMP metallopeptidase inhibitor 2 |
| XRCC5 | X-ray repair complementing defective repair in Chinese hamster cells 5 (double-strand-break rejoining) |
|  |  |
| **NEGATIVE REGULATION OF BIOLOGICAL PROCESS** | |
| ARG2 | arginase, type II |
| C1D | C1D nuclear receptor co-repressor |
| CALR | calreticulin |
| CTNNB1 | catenin (cadherin-associated protein), beta 1, 88kDa |
| DAD1 | defender against cell death 1 |
| HDAC7 | histone deacetylase 7 |
| HMGB1 | high-mobility group box 1; high-mobility group box 1-like 10 |
| ID2 | inhibitor of DNA binding 2, dominant negative helix-loop-helix protein |
| IL6R | interleukin 6 receptor |
| LRRFIP1 | leucine rich repeat (in FLII) interacting protein 1 |
| NCOR2 | nuclear receptor co-repressor 2 |
| NR2C1 | nuclear receptor subfamily 2, group C, member 1 |
| PTK2 | PTK2 protein tyrosine kinase 2 |
| RAC1 | ras-related C3 botulinum toxin substrate 1 (rho family, small GTP binding protein Rac1) |
| RTN4 | reticulon 4 |
| SOD2 | superoxide dismutase 2, mitochondrial |
| STAT3 | signal transducer and activator of transcription 3 (acute-phase response factor) |
| TIMP2 | TIMP metallopeptidase inhibitor 2 |
| XRCC5 | X-ray repair complementing defective repair in Chinese hamster cells 5 (double-strand-break rejoining) |
|  |  |
| **REGULATION OF TRANSCRIPTION FROM RNA POLYMERASE II PROMOTER** | |
| CALR | calreticulin |
| CTNNB1 | catenin (cadherin-associated protein), beta 1, 88kDa |
| EP300 | E1A binding protein p300 |
| HMGB1 | high-mobility group box 1; high-mobility group box 1-like 10 |
| ID2 | inhibitor of DNA binding 2, dominant negative helix-loop-helix protein |
| LRRFIP1 | leucine rich repeat (in FLII) interacting protein 1 |
| NCOR2 | nuclear receptor co-repressor 2 |
| NR2C1 | nuclear receptor subfamily 2, group C, member 1 |
| PLAGL1 | pleiomorphic adenoma gene-like 1 |
| SOD2 | superoxide dismutase 2, mitochondrial |
| STAT3 | signal transducer and activator of transcription 3 (acute-phase response factor) |
| TFE3 | transcription factor binding to IGHM enhancer 3 |
|  |  |
| **NEUROGENESIS** | |
| CALR | calreticulin |
| CTNNB1 | catenin (cadherin-associated protein), beta 1, 88kDa |
| NRCAM | neuronal cell adhesion molecule |
| NRP2 | neuropilin 2 |
| PTK2 | PTK2 protein tyrosine kinase 2 |
| RAC1 | ras-related C3 botulinum toxin substrate 1 (rho family, small GTP binding protein Rac1) |
| RTN4 | reticulon 4 |
| SOD2 | superoxide dismutase 2, mitochondrial |
| STAT3 | signal transducer and activator of transcription 3 (acute-phase response factor) |
| TIMP2 | TIMP metallopeptidase inhibitor 2 |
| XRCC5 | X-ray repair complementing defective repair in Chinese hamster cells 5 (double-strand-break rejoining) |
|  |  |
| **NERVOUS SYSTEM DEVELOPMENT** | |
| CALR | calreticulin |
| CTNNB1 | catenin (cadherin-associated protein), beta 1, 88kDa |
| EP300 | E1A binding protein p300 |
| FMR1 | fragile X mental retardation 1 |
| MEF2D | myocyte enhancer factor 2D |
| NRCAM | neuronal cell adhesion molecule |
| NRP2 | neuropilin 2 |
| PTK2 | PTK2 protein tyrosine kinase 2 |
| RAC1 | ras-related C3 botulinum toxin substrate 1 (rho family, small GTP binding protein Rac1) |
| RTN4 | reticulon 4 |
| STAT3 | signal transducer and activator of transcription 3 (acute-phase response factor) |
| SOD2 | superoxide dismutase 2, mitochondrial |
| TIMP2 | TIMP metallopeptidase inhibitor 2 |
| XRCC5 | X-ray repair complementing defective repair in Chinese hamster cells 5 (double-strand-break rejoining) |
|  |  |
| **NEGATIVE REGULATION OF CELLULAR PROCESS** | |
| C1D | C1D nuclear receptor co-repressor |
| CALR | calreticulin |
| CTNNB1 | catenin (cadherin-associated protein), beta 1, 88kDa |
| DAD1 | defender against cell death 1 |
| HMGB1 | high-mobility group box 1; high-mobility group box 1-like 10 |
| HDAC7 | histone deacetylase 7 |
| ID2 | inhibitor of DNA binding 2, dominant negative helix-loop-helix protein |
| LRRFIP1 | leucine rich repeat (in FLII) interacting protein 1 |
| NCOR2 | nuclear receptor co-repressor 2 |
| NR2C1 | nuclear receptor subfamily 2, group C, member 1 |
| PTK2 | PTK2 protein tyrosine kinase 2 |
| RAC1 | ras-related C3 botulinum toxin substrate 1 (rho family, small GTP binding protein Rac1) |
| RTN4 | reticulon 4 |
| STAT3 | signal transducer and activator of transcription 3 (acute-phase response factor) |
| SOD2 | superoxide dismutase 2, mitochondrial |
| TIMP2 | TIMP metallopeptidase inhibitor 2 |
| XRCC5 | X-ray repair complementing defective repair in Chinese hamster cells 5 (double-strand-break rejoining) |
|  |  |
| **REGULATION OF MULTICELLULAR ORGANISMAL PROCESS** | |
| ARG2 | arginase, type II |
| CALR | calreticulin |
| CTNNB1 | catenin (cadherin-associated protein), beta 1, 88kDa |
| HDAC7 | histone deacetylase 7 |
| ID2 | inhibitor of DNA binding 2, dominant negative helix-loop-helix protein |
| IL6R | interleukin 6 receptor |
| NRCAM | neuronal cell adhesion molecule |
| PTK2 | PTK2 protein tyrosine kinase 2 |
| RTN4 | reticulon 4 |
| STAT3 | signal transducer and activator of transcription 3 (acute-phase response factor) |
| TIMP2 | TIMP metallopeptidase inhibitor 2 |
| TFE3 | transcription factor binding to IGHM enhancer 3 |
| XRCC5 | X-ray repair complementing defective repair in Chinese hamster cells 5 (double-strand-break rejoining) |
|  |  |
| **NEGATIVE REGULATION OF MACROMOLECULE BIOSYNTHETIC PROCESS** | |
| C1D | C1D nuclear receptor co-repressor |
| CALR | calreticulin |
| CTNNB1 | catenin (cadherin-associated protein), beta 1, 88kDa |
| HMGB1 | high-mobility group box 1; high-mobility group box 1-like 10 |
| ID2 | inhibitor of DNA binding 2, dominant negative helix-loop-helix protein |
| IL6R | interleukin 6 receptor |
| LRRFIP1 | leucine rich repeat (in FLII) interacting protein 1 |
| NCOR2 | nuclear receptor co-repressor 2 |
| NR2C1 | nuclear receptor subfamily 2, group C, member 1 |
| STAT3 | signal transducer and activator of transcription 3 (acute-phase response factor) |
|  |  |
| **REGULATION OF CELLULAR PROCESS** | |
| ARG2 | arginase, type II |
| C1D | C1D nuclear receptor co-repressor |
| CALR | calreticulin |
| CREM | cAMP responsive element modulator |
| CTNNB1 | catenin (cadherin-associated protein), beta 1, 88kDa |
| CCNC | cyclin C |
| DAD1 | defender against cell death 1 |
| DBNL | drebrin-like |
| EP300 | E1A binding protein p300 |
| GDI1 | GDP dissociation inhibitor 1 |
| HMGB1 | high-mobility group box 1; high-mobility group box 1-like 10 |
| HDAC7 | histone deacetylase 7 |
| ID2 | inhibitor of DNA binding 2, dominant negative helix-loop-helix protein |
| IL6R | interleukin 6 receptor |
| KDR | kinase insert domain receptor (a type III receptor tyrosine kinase) |
| LEPR | leptin receptor |
| LRRFIP1 | leucine rich repeat (in FLII) interacting protein 1 |
| MEF2D | myocyte enhancer factor 2D |
| NRCAM | neuronal cell adhesion molecule |
| NCOR2 | nuclear receptor co-repressor 2 |
| NR2C1 | nuclear receptor subfamily 2, group C, member 1 |
| PRDX4 | peroxiredoxin 4 |
| PLAGL1 | pleiomorphic adenoma gene-like 1 |
| PTK2 | PTK2 protein tyrosine kinase 2 |
| RAB7A | RAB7A, member RAS oncogene family |
| RAC1 | ras-related C3 botulinum toxin substrate 1 (rho family, small GTP binding protein Rac1) |
| RTN4 | reticulon 4 |
| STAT3 | signal transducer and activator of transcription 3 (acute-phase response factor) |
| SOD2 | superoxide dismutase 2, mitochondrial |
| TXN | thioredoxin |
| TIMP2 | TIMP metallopeptidase inhibitor 2 |
| TFE3 | transcription factor binding to IGHM enhancer 3 |
| TNFSF10 | tumor necrosis factor (ligand) superfamily, member 10 |
| VCAM1 | vascular cell adhesion molecule 1 |
| XRCC5 | X-ray repair complementing defective repair in Chinese hamster cells 5 (double-strand-break rejoining) |

**Supplemental Table 3**

**GO ANALYSIS OF GENES CORRELATED IN SUICIDE GROUP**

| **REGULATION OF CELLULAR COMPONENT**  **BIOGENESIS** | |
| --- | --- |
| HMGB1 | high-mobility group box 1; high-mobility group box 1-like 10 |
| SNAP25 | synaptosomal-associated protein, 25kDa |
| UBC | ubiquitin C |
|  |  |
| **REGULATION OF CELLULAR COMPONENT ORGANIZATION** | |
| HMGB1 | high-mobility group box 1; high-mobility group box 1-like 10 |
| RTN4 | reticulon 4 |
| SNAP25 | synaptosomal-associated protein, 25kDa |
| UBC | ubiquitin C |
|  |  |
| **REGULATION OF SYNAPTOGENESIS** | |
| SNAP25 | synaptosomal-associated protein, 25kDa |
| UBC | ubiquitin C |
|  |  |
| **REGULATION OF NERVOUS SYSTEM DEVELOPMENT** | |
| RTN4 | reticulon 4 |
| SNAP25 | synaptosomal-associated protein, 25kDa |
| UBC | ubiquitin C |
|  |  |
| **REGULATION OF SYNAPSE ORGANIZATION** | |
| SNAP25 | synaptosomal-associated protein, 25kDa |
| UBC | ubiquitin C |
|  |  |
| **REGULATION OF SYNAPSE STRUCTURE AND ACTIVITY** | |
| SNAP25 | synaptosomal-associated protein, 25kDa |
| UBC | ubiquitin C |
|  |  |
| **REGULATION OF DEVELOPMENTAL PROCESS** | |
| GNAS | GNAS complex locus |
| RTN4 | reticulon 4 |
| SNAP25 | synaptosomal-associated protein, 25kDa |
| UBC | ubiquitin C |
|  |  |
| **SYNAPTIC TRANSMISSION** | |
| DTNA | dystrobrevin, alpha |
| SNAP25 | synaptosomal-associated protein, 25kDa |
| UBC | ubiquitin C |
|  |  |
| **REGULATION OF DEVELOPMENTAL GROWTH** | |
| RTN4 | reticulon 4 |
| UBC | ubiquitin C |
|  |  |
| **NITROGEN COMPOUND BIOSYNTHETIC PROCESS** | |
| ATP13A5 | ATPase type 13A5 |
| ATP1B1 | ATPase, Na+/K+ transporting, beta 1 polypeptide |
| GLUL | glutamate-ammonia ligase (glutamine synthetase) |

**Supplemental Table 3**

**GO ANALYSIS OF GENES CORRELATED IN SUICIDE GROUP**

| **REGULATION OF CELLULAR COMPONENT**  **BIOGENESIS** | |
| --- | --- |
| HMGB1 | high-mobility group box 1; high-mobility group box 1-like 10 |
| SNAP25 | synaptosomal-associated protein, 25kDa |
| UBC | ubiquitin C |
|  |  |
| **REGULATION OF CELLULAR COMPONENT ORGANIZATION** | |
| HMGB1 | high-mobility group box 1; high-mobility group box 1-like 10 |
| RTN4 | reticulon 4 |
| SNAP25 | synaptosomal-associated protein, 25kDa |
| UBC | ubiquitin C |
|  |  |
| **REGULATION OF SYNAPTOGENESIS** | |
| SNAP25 | synaptosomal-associated protein, 25kDa |
| UBC | ubiquitin C |
|  |  |
| **REGULATION OF NERVOUS SYSTEM DEVELOPMENT** | |
| RTN4 | reticulon 4 |
| SNAP25 | synaptosomal-associated protein, 25kDa |
| UBC | ubiquitin C |
|  |  |
| **REGULATION OF SYNAPSE ORGANIZATION** | |
| SNAP25 | synaptosomal-associated protein, 25kDa |
| UBC | ubiquitin C |
|  |  |
| **REGULATION OF SYNAPSE STRUCTURE AND ACTIVITY** | |
| SNAP25 | synaptosomal-associated protein, 25kDa |
| UBC | ubiquitin C |
|  |  |
| **REGULATION OF DEVELOPMENTAL PROCESS** | |
| GNAS | GNAS complex locus |
| RTN4 | reticulon 4 |
| SNAP25 | synaptosomal-associated protein, 25kDa |
| UBC | ubiquitin C |
|  |  |
| **SYNAPTIC TRANSMISSION** | |
| DTNA | dystrobrevin, alpha |
| SNAP25 | synaptosomal-associated protein, 25kDa |
| UBC | ubiquitin C |
|  |  |
| **REGULATION OF DEVELOPMENTAL GROWTH** | |
| RTN4 | reticulon 4 |
| UBC | ubiquitin C |
|  |  |
| **NITROGEN COMPOUND BIOSYNTHETIC PROCESS** | |
| ATP13A5 | ATPase type 13A5 |
| ATP1B1 | ATPase, Na+/K+ transporting, beta 1 polypeptide |
| GLUL | glutamate-ammonia ligase (glutamine synthetase) |
