# Supplementary material for: Molecular Pathway Reconstruction and Analysis of Disturbed Gene Expression in Depressed Individuals Who Died by Suicide
Source: PLoS One. 2012 Oct 22;7(10):e47581. doi: 10.1371/journal.pone.0047581 (PMC3478292; doi:10.1371/journal.pone.0047581)
Supplement: Table S2 — GO analysis of DEGS from pathway analysis (largest network). (DOCX) [file pone.0047581.s002.docx]

| Table S2 |  |
| --- | --- |

| **REGULATION OF CELL DIFFERENTIATION** | |
| --- | --- |
| CALR | calreticulin |
| CTNNB1 | catenin (cadherin-associated protein), beta 1, 88kDa |
| HDAC7 | histone deacetylase 7 |
| ID2 | inhibitor of DNA binding 2, dominant negative helix-loop-helix protein |
| IL6R | interleukin 6 receptor |
| NRCAM | neuronal cell adhesion molecule |
| PTK2 | PTK2 protein tyrosine kinase 2 |
| RTN4 | reticulon 4 |
| SOD2 | superoxide dismutase 2, mitochondrial |
| TFE3 | transcription factor binding to IGHM enhancer 3 |
| TIMP2 | TIMP metallopeptidase inhibitor 2 |
| XRCC5 | X-ray repair complementing defective repair in Chinese hamster cells 5 (double-strand-break rejoining) |
|  |  |
| **REGULATION OF DEVELOPMENTAL PROCESS** | |
| CALR | calreticulin |
| CTNNB1 | catenin (cadherin-associated protein), beta 1, 88kDa |
| HDAC7 | histone deacetylase 7 |
| ID2 | inhibitor of DNA binding 2, dominant negative helix-loop-helix protein |
| IL6R | interleukin 6 receptor |
| KDR | kinase insert domain receptor (a type III receptor tyrosine kinase) |
| NRCAM | neuronal cell adhesion molecule |
| PTK2 | PTK2 protein tyrosine kinase 2 |
| RTN4 | reticulon 4 |
| SOD2 | superoxide dismutase 2, mitochondrial |
| TFE3 | transcription factor binding to IGHM enhancer 3 |
| TIMP2 | TIMP metallopeptidase inhibitor 2 |
| XRCC5 | X-ray repair complementing defective repair in Chinese hamster cells 5 (double-strand-break rejoining) |
|  |  |
| **NEGATIVE REGULATION OF BIOLOGICAL PROCESS** | |
| ARG2 | arginase, type II |
| C1D | C1D nuclear receptor co-repressor |
| CALR | calreticulin |
| CTNNB1 | catenin (cadherin-associated protein), beta 1, 88kDa |
| DAD1 | defender against cell death 1 |
| HDAC7 | histone deacetylase 7 |
| HMGB1 | high-mobility group box 1; high-mobility group box 1-like 10 |
| ID2 | inhibitor of DNA binding 2, dominant negative helix-loop-helix protein |
| IL6R | interleukin 6 receptor |
| LRRFIP1 | leucine rich repeat (in FLII) interacting protein 1 |
| NCOR2 | nuclear receptor co-repressor 2 |
| NR2C1 | nuclear receptor subfamily 2, group C, member 1 |
| PTK2 | PTK2 protein tyrosine kinase 2 |
| RAC1 | ras-related C3 botulinum toxin substrate 1 (rho family, small GTP binding protein Rac1) |
| RTN4 | reticulon 4 |
| SOD2 | superoxide dismutase 2, mitochondrial |
| STAT3 | signal transducer and activator of transcription 3 (acute-phase response factor) |
| TIMP2 | TIMP metallopeptidase inhibitor 2 |
| XRCC5 | X-ray repair complementing defective repair in Chinese hamster cells 5 (double-strand-break rejoining) |
|  |  |
| **REGULATION OF TRANSCRIPTION FROM RNA POLYMERASE II PROMOTER** | |
| CALR | calreticulin |
| CTNNB1 | catenin (cadherin-associated protein), beta 1, 88kDa |
| EP300 | E1A binding protein p300 |
| HMGB1 | high-mobility group box 1; high-mobility group box 1-like 10 |
| ID2 | inhibitor of DNA binding 2, dominant negative helix-loop-helix protein |
| LRRFIP1 | leucine rich repeat (in FLII) interacting protein 1 |
| NCOR2 | nuclear receptor co-repressor 2 |
| NR2C1 | nuclear receptor subfamily 2, group C, member 1 |
| PLAGL1 | pleiomorphic adenoma gene-like 1 |
| SOD2 | superoxide dismutase 2, mitochondrial |
| STAT3 | signal transducer and activator of transcription 3 (acute-phase response factor) |
| TFE3 | transcription factor binding to IGHM enhancer 3 |
|  |  |
| **NEUROGENESIS** | |
| CALR | calreticulin |
| CTNNB1 | catenin (cadherin-associated protein), beta 1, 88kDa |
| NRCAM | neuronal cell adhesion molecule |
| NRP2 | neuropilin 2 |
| PTK2 | PTK2 protein tyrosine kinase 2 |
| RAC1 | ras-related C3 botulinum toxin substrate 1 (rho family, small GTP binding protein Rac1) |
| RTN4 | reticulon 4 |
| SOD2 | superoxide dismutase 2, mitochondrial |
| STAT3 | signal transducer and activator of transcription 3 (acute-phase response factor) |
| TIMP2 | TIMP metallopeptidase inhibitor 2 |
| XRCC5 | X-ray repair complementing defective repair in Chinese hamster cells 5 (double-strand-break rejoining) |
|  |  |
| **NERVOUS SYSTEM DEVELOPMENT** | |
| CALR | calreticulin |
| CTNNB1 | catenin (cadherin-associated protein), beta 1, 88kDa |
| EP300 | E1A binding protein p300 |
| FMR1 | fragile X mental retardation 1 |
| MEF2D | myocyte enhancer factor 2D |
| NRCAM | neuronal cell adhesion molecule |
| NRP2 | neuropilin 2 |
| PTK2 | PTK2 protein tyrosine kinase 2 |
| RAC1 | ras-related C3 botulinum toxin substrate 1 (rho family, small GTP binding protein Rac1) |
| RTN4 | reticulon 4 |
| STAT3 | signal transducer and activator of transcription 3 (acute-phase response factor) |
| SOD2 | superoxide dismutase 2, mitochondrial |
| TIMP2 | TIMP metallopeptidase inhibitor 2 |
| XRCC5 | X-ray repair complementing defective repair in Chinese hamster cells 5 (double-strand-break rejoining) |
|  |  |
| **NEGATIVE REGULATION OF CELLULAR PROCESS** | |
| C1D | C1D nuclear receptor co-repressor |
| CALR | calreticulin |
| CTNNB1 | catenin (cadherin-associated protein), beta 1, 88kDa |
| DAD1 | defender against cell death 1 |
| HMGB1 | high-mobility group box 1; high-mobility group box 1-like 10 |
| HDAC7 | histone deacetylase 7 |
| ID2 | inhibitor of DNA binding 2, dominant negative helix-loop-helix protein |
| LRRFIP1 | leucine rich repeat (in FLII) interacting protein 1 |
| NCOR2 | nuclear receptor co-repressor 2 |
| NR2C1 | nuclear receptor subfamily 2, group C, member 1 |
| PTK2 | PTK2 protein tyrosine kinase 2 |
| RAC1 | ras-related C3 botulinum toxin substrate 1 (rho family, small GTP binding protein Rac1) |
| RTN4 | reticulon 4 |
| STAT3 | signal transducer and activator of transcription 3 (acute-phase response factor) |
| SOD2 | superoxide dismutase 2, mitochondrial |
| TIMP2 | TIMP metallopeptidase inhibitor 2 |
| XRCC5 | X-ray repair complementing defective repair in Chinese hamster cells 5 (double-strand-break rejoining) |
|  |  |
| **REGULATION OF MULTICELLULAR ORGANISMAL PROCESS** | |
| ARG2 | arginase, type II |
| CALR | calreticulin |
| CTNNB1 | catenin (cadherin-associated protein), beta 1, 88kDa |
| HDAC7 | histone deacetylase 7 |
| ID2 | inhibitor of DNA binding 2, dominant negative helix-loop-helix protein |
| IL6R | interleukin 6 receptor |
| NRCAM | neuronal cell adhesion molecule |
| PTK2 | PTK2 protein tyrosine kinase 2 |
| RTN4 | reticulon 4 |
| STAT3 | signal transducer and activator of transcription 3 (acute-phase response factor) |
| TIMP2 | TIMP metallopeptidase inhibitor 2 |
| TFE3 | transcription factor binding to IGHM enhancer 3 |
| XRCC5 | X-ray repair complementing defective repair in Chinese hamster cells 5 (double-strand-break rejoining) |
|  |  |
| **NEGATIVE REGULATION OF MACROMOLECULE BIOSYNTHETIC PROCESS** | |
| C1D | C1D nuclear receptor co-repressor |
| CALR | calreticulin |
| CTNNB1 | catenin (cadherin-associated protein), beta 1, 88kDa |
| HMGB1 | high-mobility group box 1; high-mobility group box 1-like 10 |
| ID2 | inhibitor of DNA binding 2, dominant negative helix-loop-helix protein |
| IL6R | interleukin 6 receptor |
| LRRFIP1 | leucine rich repeat (in FLII) interacting protein 1 |
| NCOR2 | nuclear receptor co-repressor 2 |
| NR2C1 | nuclear receptor subfamily 2, group C, member 1 |
| STAT3 | signal transducer and activator of transcription 3 (acute-phase response factor) |
|  |  |
| **REGULATION OF CELLULAR PROCESS** | |
| ARG2 | arginase, type II |
| C1D | C1D nuclear receptor co-repressor |
| CALR | calreticulin |
| CREM | cAMP responsive element modulator |
| CTNNB1 | catenin (cadherin-associated protein), beta 1, 88kDa |
| CCNC | cyclin C |
| DAD1 | defender against cell death 1 |
| DBNL | drebrin-like |
| EP300 | E1A binding protein p300 |
| GDI1 | GDP dissociation inhibitor 1 |
| HMGB1 | high-mobility group box 1; high-mobility group box 1-like 10 |
| HDAC7 | histone deacetylase 7 |
| ID2 | inhibitor of DNA binding 2, dominant negative helix-loop-helix protein |
| IL6R | interleukin 6 receptor |
| KDR | kinase insert domain receptor (a type III receptor tyrosine kinase) |
| LEPR | leptin receptor |
| LRRFIP1 | leucine rich repeat (in FLII) interacting protein 1 |
| MEF2D | myocyte enhancer factor 2D |
| NRCAM | neuronal cell adhesion molecule |
| NCOR2 | nuclear receptor co-repressor 2 |
| NR2C1 | nuclear receptor subfamily 2, group C, member 1 |
| PRDX4 | peroxiredoxin 4 |
| PLAGL1 | pleiomorphic adenoma gene-like 1 |
| PTK2 | PTK2 protein tyrosine kinase 2 |
| RAB7A | RAB7A, member RAS oncogene family |
| RAC1 | ras-related C3 botulinum toxin substrate 1 (rho family, small GTP binding protein Rac1) |
| RTN4 | reticulon 4 |
| STAT3 | signal transducer and activator of transcription 3 (acute-phase response factor) |
| SOD2 | superoxide dismutase 2, mitochondrial |
| TXN | thioredoxin |
| TIMP2 | TIMP metallopeptidase inhibitor 2 |
| TFE3 | transcription factor binding to IGHM enhancer 3 |
| TNFSF10 | tumor necrosis factor (ligand) superfamily, member 10 |
| VCAM1 | vascular cell adhesion molecule 1 |
| XRCC5 | X-ray repair complementing defective repair in Chinese hamster cells 5 (double-strand-break rejoining) |
