# Supplementary material for: Molecular Pathway Reconstruction and Analysis of Disturbed Gene Expression in Depressed Individuals Who Died by Suicide
Source: PLoS One. 2012 Oct 22;7(10):e47581. doi: 10.1371/journal.pone.0047581 (PMC3478292; doi:10.1371/journal.pone.0047581)
Supplement: Table S3 — GO analysis of genes correlated in suicide group. (DOCX) [file pone.0047581.s003.docx]

Table S3

| **REGULATION OF CELLULAR COMPONENT**  **BIOGENESIS** | |
| --- | --- |
| HMGB1 | high-mobility group box 1; high-mobility group box 1-like 10 |
| SNAP25 | synaptosomal-associated protein, 25kDa |
| UBC | ubiquitin C |
|  |  |
| **REGULATION OF CELLULAR COMPONENT ORGANIZATION** | |
| HMGB1 | high-mobility group box 1; high-mobility group box 1-like 10 |
| RTN4 | reticulon 4 |
| SNAP25 | synaptosomal-associated protein, 25kDa |
| UBC | ubiquitin C |
|  |  |
| **REGULATION OF SYNAPTOGENESIS** | |
| SNAP25 | synaptosomal-associated protein, 25kDa |
| UBC | ubiquitin C |
|  |  |
| **REGULATION OF NERVOUS SYSTEM DEVELOPMENT** | |
| RTN4 | reticulon 4 |
| SNAP25 | synaptosomal-associated protein, 25kDa |
| UBC | ubiquitin C |
|  |  |
| **REGULATION OF SYNAPSE ORGANIZATION** | |
| SNAP25 | synaptosomal-associated protein, 25kDa |
| UBC | ubiquitin C |
|  |  |
| **REGULATION OF SYNAPSE STRUCTURE AND ACTIVITY** | |
| SNAP25 | synaptosomal-associated protein, 25kDa |
| UBC | ubiquitin C |
|  |  |
| **REGULATION OF DEVELOPMENTAL PROCESS** | |
| GNAS | GNAS complex locus |
| RTN4 | reticulon 4 |
| SNAP25 | synaptosomal-associated protein, 25kDa |
| UBC | ubiquitin C |
|  |  |
| **SYNAPTIC TRANSMISSION** | |
| DTNA | dystrobrevin, alpha |
| SNAP25 | synaptosomal-associated protein, 25kDa |
| UBC | ubiquitin C |
|  |  |
| **REGULATION OF DEVELOPMENTAL GROWTH** | |
| RTN4 | reticulon 4 |
| UBC | ubiquitin C |
|  |  |
| **NITROGEN COMPOUND BIOSYNTHETIC PROCESS** | |
| ATP13A5 | ATPase type 13A5 |
| ATP1B1 | ATPase, Na+/K+ transporting, beta 1 polypeptide |
| GLUL | glutamate-ammonia ligase (glutamine synthetase) |
